# Supplementary material for: iPSC-derived models of PACS1 syndrome reveal transcriptional and functional deficits in neuron activity
Source: Nat Commun. 2024 Jan 27;15:827. doi: 10.1038/s41467-024-44989-7 (PMC10821916; doi:10.1038/s41467-024-44989-7)
Supplement: Supplementary file 1 — Supplementary Information [file 41467_2024_44989_MOESM1_ESM.pdf]

Supplementary materials for

iPSC-derived models of PACS1 syndrome reveal transcriptional and functional deficits in neuron activity

Lauren Rylaarsdam, Jennifer Rakotomamonjy, Eleanor Pope, Alicia Guemez-Gamboa

This file includes:

Figures S1-S15 and legends

Table S1 and legend

Data S1 legend (separate file)

SUPPLEMENTARY FIGURES

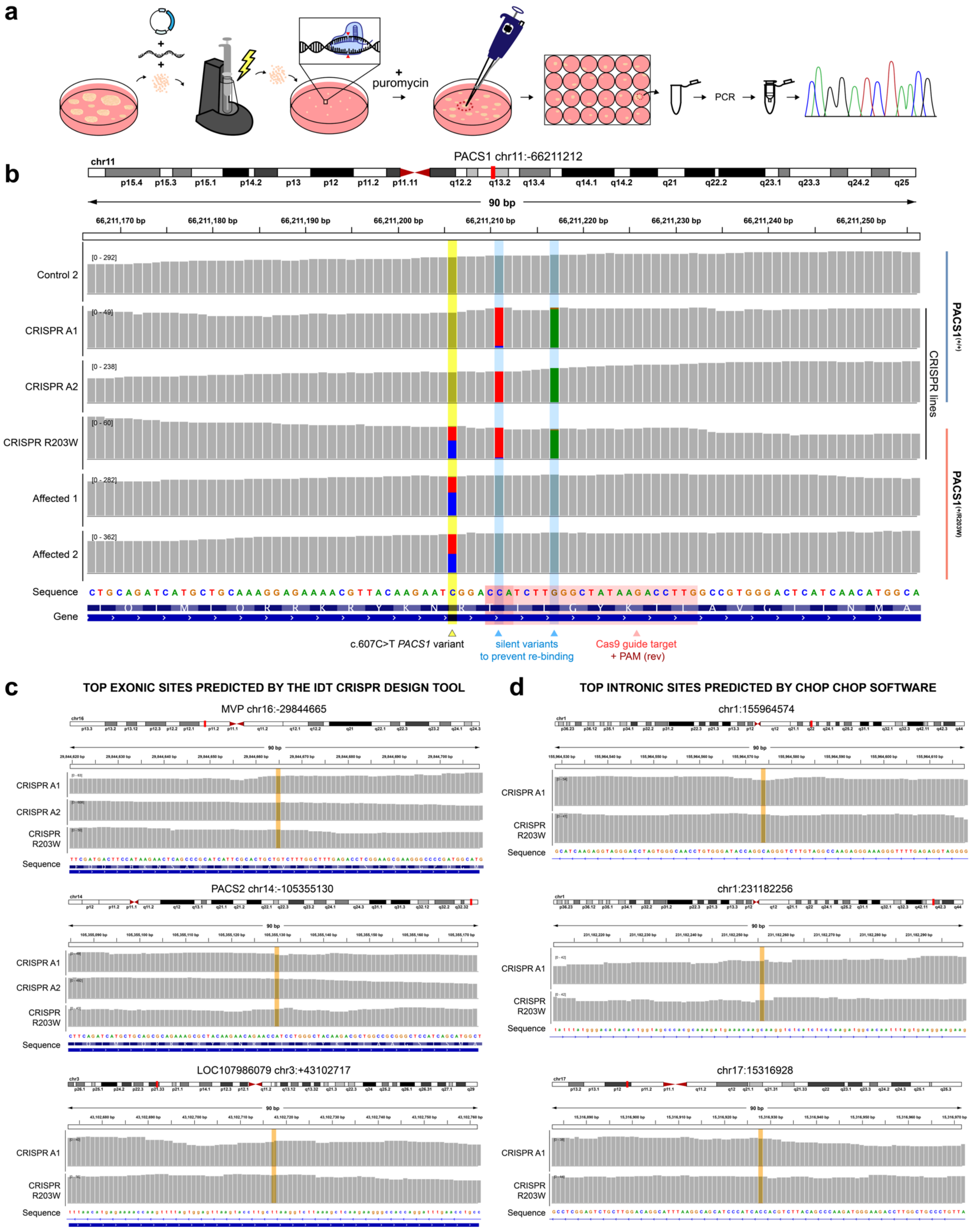

Figure S1. Generation and validation of isogenic stem cell lines. a) Induced pluripotent stem cells (iPSCs) were electroporated with a plasmid expressing Cas9, a guide targeting exon 4 of *PACS1*, and a single-stranded oligodeoxynucleotide donor containing the c.607C>T variant in addition to two silent variants (c.612C>T and c.618G>A) to prevent re-binding of the guide. **b**) Sequencing validates introduction of the heterozygous c.607C>T variant in the CRISPR R203W line - also observed in patient lines A1 and A2 (boxed in yellow) - and successful correction of the patient variant in CRISPR A1 and CRISPR A2 lines. In CRISPR-edited lines, the two downstream additional variants (boxed in blue) are the silent variants. The guide target region is boxed in red with the PAM site towards the 5' end. **c)** Top exonic off-target sites predicted by the IDT CRISPR design tool. The predicted site is highlighted in orange with the surrounding 90 bp showing no unintended edits were introduced. CRISPR A2 is missing from the final panel as it received exome sequencing instead of whole-genome sequencing, which did not cover this noncoding RNA. **d)** Top predicted intronic off-target sites predicted by CHOP CHOP software. The predicted site is highlighted in orange with the surrounding 90 bp showing no unintended edits were introduced. Not all lines are present as only CRISPR R203W and CRISPR A1 received whole-genome sequencing.

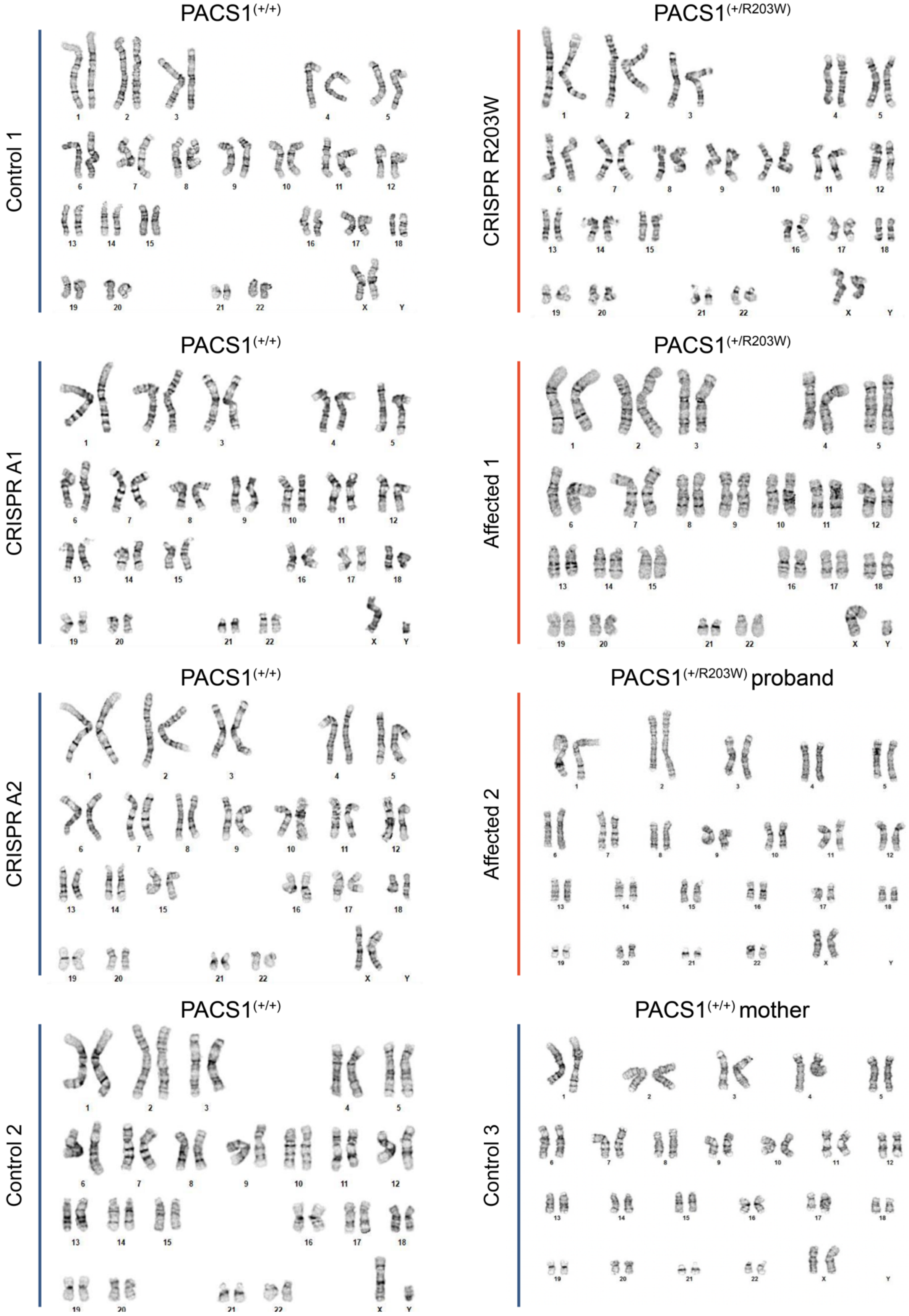

Figure S2. iPSC parent and CRISPR-edited lines display normal karyotypes. Karyotype analysis for all induced pluripotent stem cell (iPSC) lines used in this study. PACS1$^{(+/+)}$ and PACS1$^{(+/R203W)}$ lines display a normal karyotype, including CRISPR-edited lines.

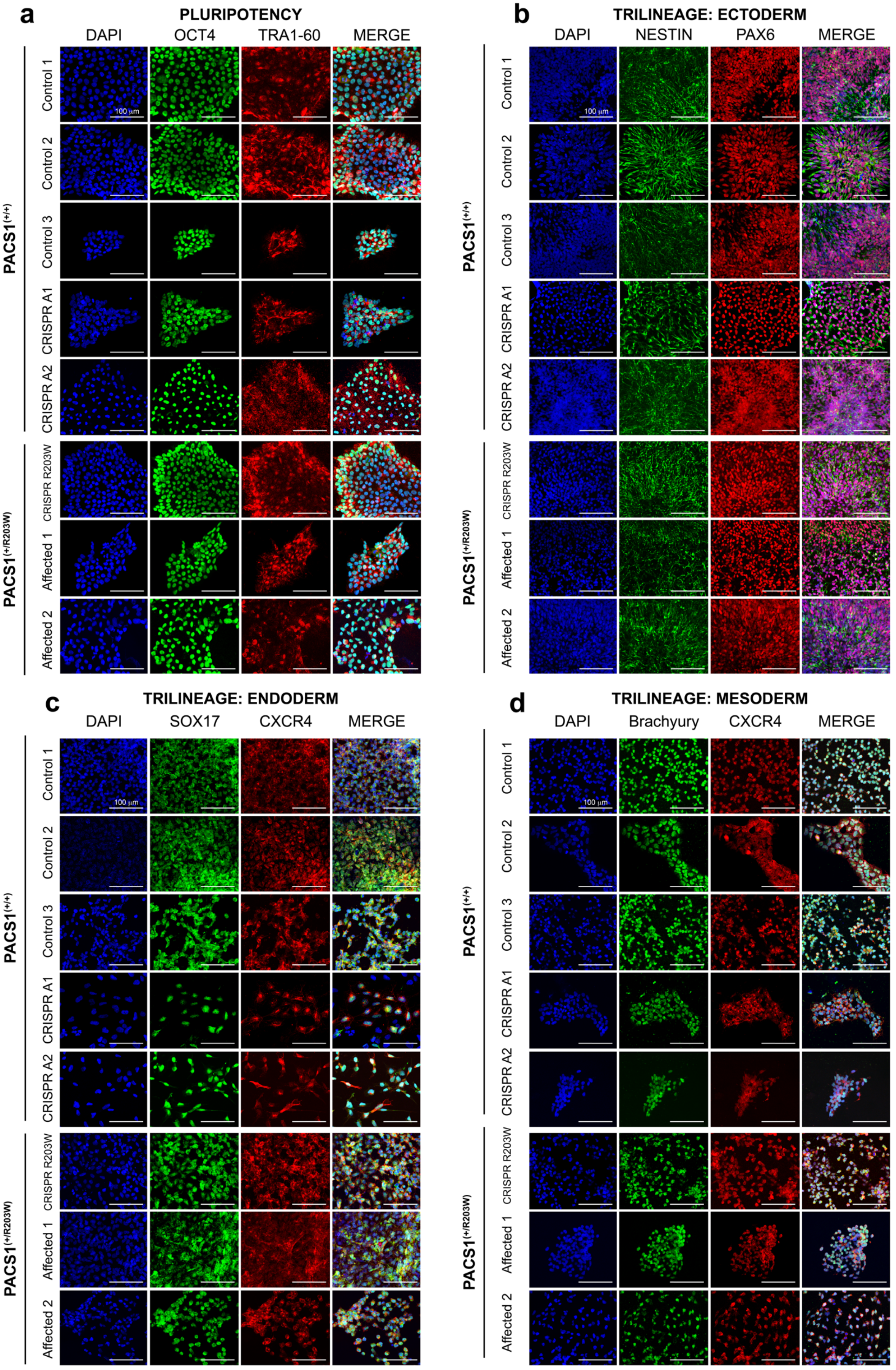
a
PLURIPOTENCY
DAPI
OCT4
TRA1-60
MERGE
Control 1
Control 2
Control 3
CRISPR A1
CRISPR A2
CRISPR R203W
Affected 1
Affected 2
PACS1(+/+)
PACS1(+/R203W)
100 µm
b
TRILINEAGE: ECTODERM
DAPI
NESTIN
PAX6
MERGE
c
TRILINEAGE: ENDODERM
DAPI
SOX17
CXCR4
MERGE
d
TRILINEAGE: MESODERM
DAPI
Brachyury
CXCR4
MERGE

Figure S3: iPSC parent and CRISPR-edited lines express pluripotent markers and have the capacity to generate all three germ layers. a) Immunohistochemistry analysis demonstrates induced pluripotent stem cell (iPSC) lines express pluripotent markers OCT4 and TRA1-60. Scale bar = 100 μm. Pluripotent capacity is further demonstrated by the ability to generate all three germ layers in **b-d. b)** iPSCs differentiated to ectodermal lineage as indicated by co-expression of PAX6 and Nestin. Scale bar = 100 μm. **c)** iPSCs directed to endodermal lineage as indicated by co-expression of SOX17 and CXCR4. Scale bar = 100 μm. **d)** iPSCs differentiated towards mesodermal lineage as indicated by co-expression of Brachyury and CXCR4. Scale bar = 100 μm.

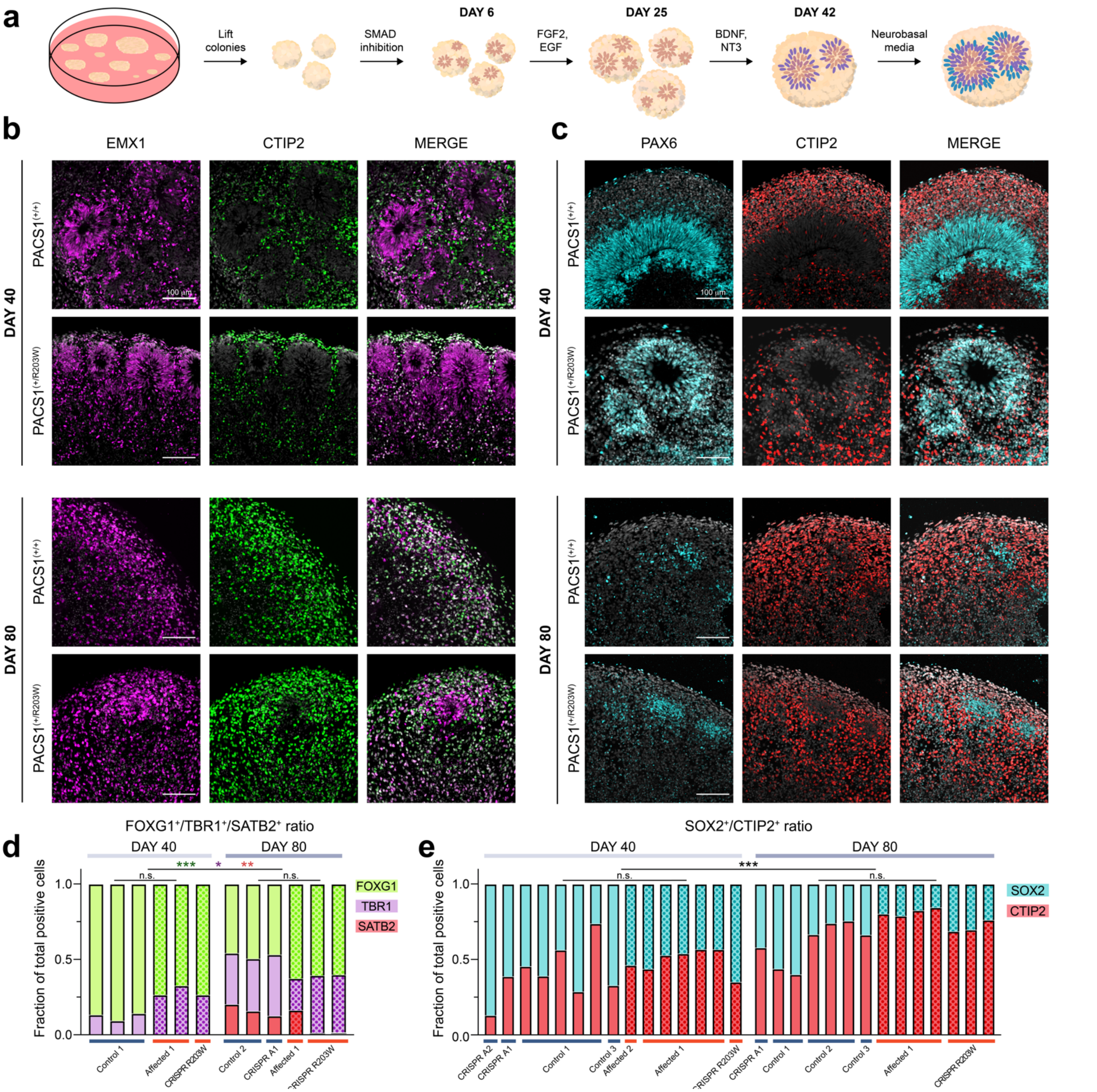

Figure S4. Generation and validation of a dorsal cortical organoid model system. **a)** Dorsal cortical organoids were generated using a growth factor program to induce neuroectoderm differentiation, proliferation, and survival. **b)** Expression patterns of cortical protein EMX1 and neuronal transcription factor CTIP2 in day 40 and 80 organoids. Scale bar = 100 µm. DAPI is in greyscale. **c)** Expression patterns of dorsal forebrain progenitor protein PAX6 and postmitotic neuron marker CTIP2 in day 40 and 80 organoids. Scale bar = 100 µm. DAPI is in greyscale. **d**) FOXG1$^+$/TBR1$^+$/SATB2$^+$ ratios shown in **Fig. 1d** by organoid. Bar shows the mean fraction of positive cells contributing to the total number of positive cells per image field. Stains are different by day (FOXG1 $F_{1,9}$=24.004, p_{adj} = 8.43E-04; TBR1 $F_{1,9}$=9.459, p_{adj} = 0.013; SATB2 H_1=9.466, p_{adj} = 0.004) but not genotype (FOXG1 $F_{1,9}$=0.061, p_{adj} =0.811; TBR1 $F_{1,9}$=1.811, p_{adj} = 0.211; SATB2 H_1 = 0.183, p_{adj}=0.669) using a two-way ANOVA unless $p < 0.05$ in a Shapiro test of normality (see **Methods**). $n = 3$ organoids for each condition (Day 40: three C1, one CRISPR R203W, and two A1 organoids from four independent differentiations; Day 80: two C2, one CRISPR A1, two CRISPR R203W, and one A1 organoids

from three independent differentiations. See **Table S1**). Data from PACS1$^{(+/R203W)}$ lines is indicated by the dot pattern. **e)** SOX2^{+}/CTIP2^{+} ratios shown in **Fig. 1e** by organoid. Bar shows the mean fraction of positive cells contributing to the total number of positive cells per image field. Mean ± SEM fraction of CTIP2^{+} or SOX2^{+} cells contributing to the total number of positive cells per field of view. The ratio was significant between time points (H_1=12.808, p_{adj} = 6.90E-04) but not genotypes (H_1=3.360, p_{adj} = 0.067). n = 7+ organoids per condition (Day 40: five C1, one C3, one CRISPR A1, one CRISPR A2, one CRISPR R203W, five A1, and one A2 organoids from four independent differentiations; Day 80: two C1, three C2, one C3, one CRISPR A1, three CRISPR R203W, and four A1 organoids from three independent differentiations. See **Table S1**). Data from PACS1$^{(+/R203W)}$ lines is indicated by the dot pattern. Source data for **d-e** can be found in the Source Data file.

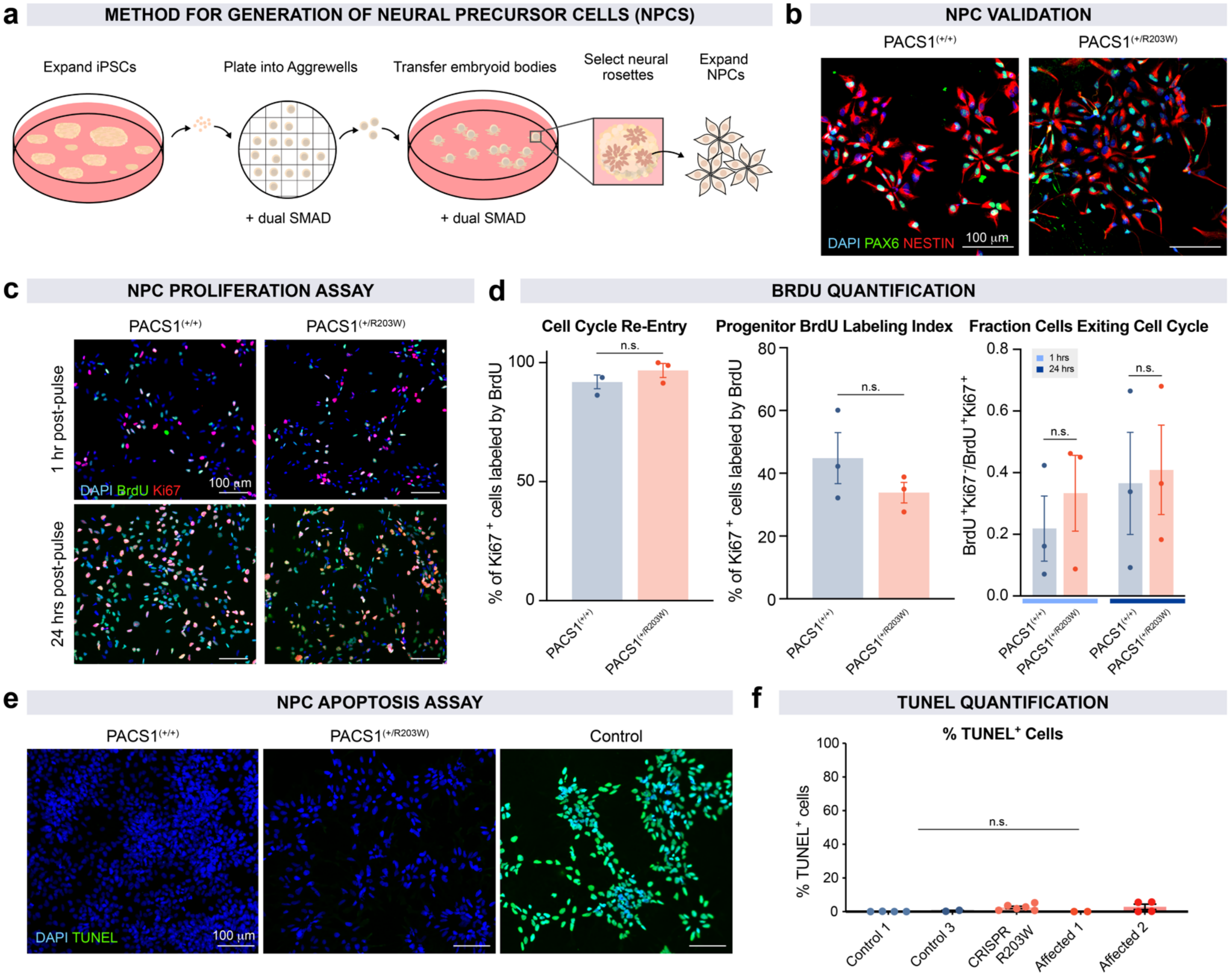

Figure S5: PACS1 p.R203W variant does not impact rates of neural precursor proliferation or apoptosis. a) Protocol for the differentiation of induced pluripotent stem cells (iPSCs) to neural precursor cells (NPCs). In brief, iPSCs are dissociated and plated in Aggrewells to generate embryoid bodies (EBs). EBs are exposed to dual SMAD inhibitors to direct ectoderm formation. Rosettes are selected and expanded. **b)** PAX6$^+$Nestin$^+$ NPCs were successfully generated in both PACS1$^{(+/+)}$ and PACS1$^{(+/R203W)}$ cell lines. Scale bar = 100 µm. **c)** Proliferation rate of NPCs was assessed by pulsing the thymidine analog BrdU and co-staining for mitotic marker Ki67. Scale bar = 100 µm. **d)** Mean ± SEM of proliferation metrics as calculated from **c**. n = 3 biological replicates from two independent experiments (one Cl, one C2, one C3, one CRISPR R203W, one A1, and one A2 coverslip average). Genotype had no effect on cell cycle re-entry ($t_{4.00}$ = -1.282, p = 0.269 using a Welch two sample two-sided t test), progenitor BrdU labeling index ($t_{2.62}$=1.253, p = 0.310 using a Welch two sample two-sided t test), or fraction of cells exiting the cell cycle (F_1=0.376, p_{adj} = 0.555 using a two-way ANOVA with a Tukey HSD correction). Source data can be found in the Source Data file. **e)** NPC apoptosis rates were assessed using a TUNEL assay. Co-localization of DAPI and TUNEL is shown in PACS1$^{(+/+)}$ and PACS1$^{(+/R203W)}$ NPCs, along with a DNAse-treated positive control. Scale bar = 100 µm. **f)** Quantification of the TUNEL assay in **e** shows a very low rate of TUNEL$^+$ cells in each cell line tested. Dots are coverslip image values from n = 3 PACS1$^{(+/+)}$ lines (two C1 and one C3) and n = 6 PACS1$^{(+/R203W)}$ lines (three CRISPR R203W, one A1, and two A2) from three independent experiments. Genotype has no effect on the incidence of TUNEL$^+$ cells (H_1= 1.361, p = 0.243 using a one-sided Kruskal Wallis test as $p_{shapiro}$ = 0.0174). Source data can be found in the Source Data file.

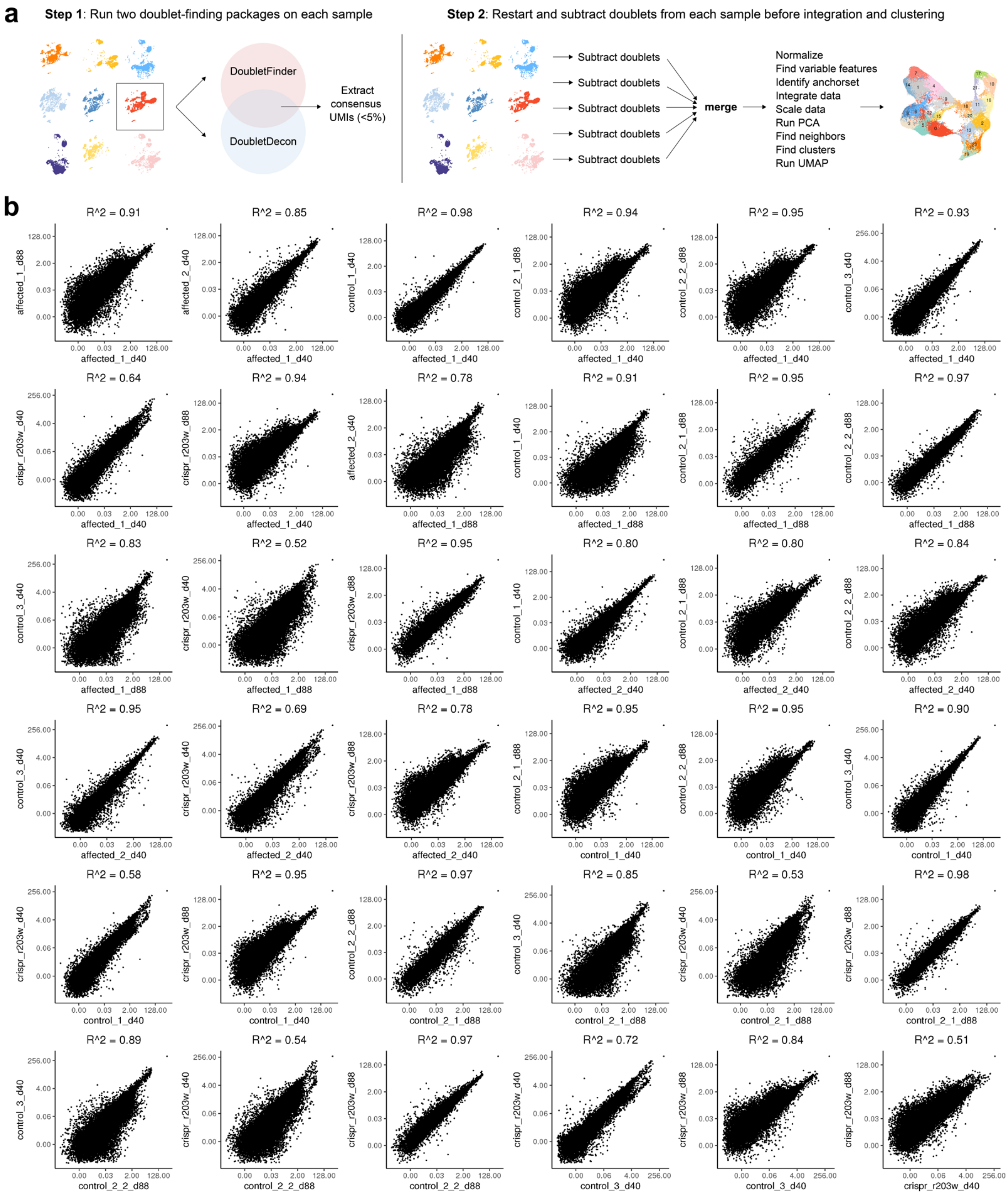

Figure S6. Single-cell RNA sequencing in day 40 and 88 organoids. a) Initial sample processing schematic. First, each sample was individually constructed, and two separate packages were utilized to identify doublets. Consensus doublet barcodes were removed, and samples were then all processed in parallel. **b)** Pearson correlation of gene expression between all sample combination pairs from nine organoids total across four independent differentiations (Day 40: one C1, one C3, one CRISPR R203W, one A1, and one A2 organoid; Day 88: two C2, one CRISPR R203W, and one A1 organoid. See **Table S1**).

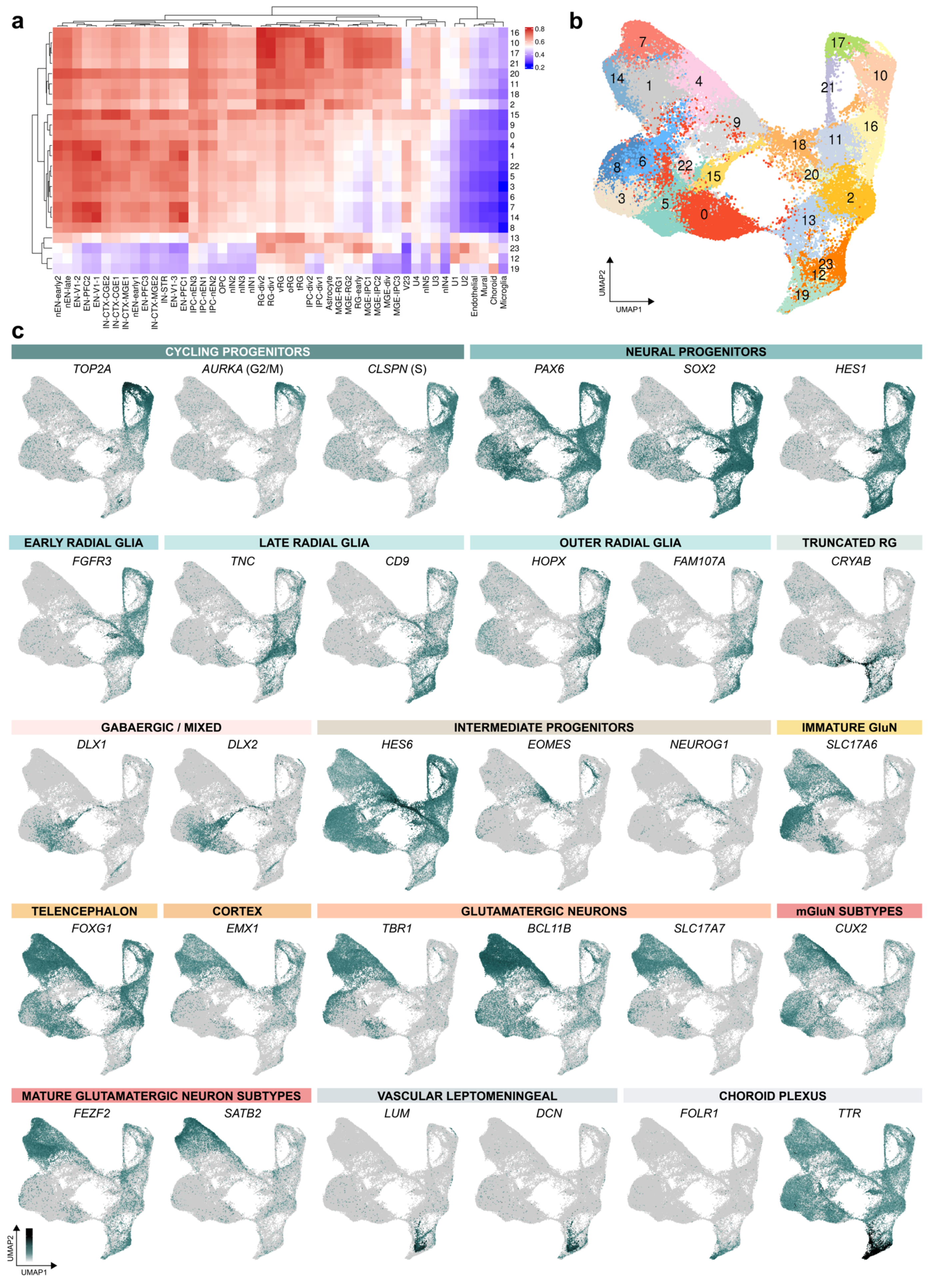
a
b
c
UMAP2
UMAP1
16
10
17
21
20
11
18
2
15
9
0
4
1
22
5
3
6
7
14
8
13
23
12
19
nEN-early2
nEN-late
EN-V1-2
EN-PFC2
EN-V1-1
IN-CTX-CGE2
IN-CTX-CGE1
IN-CTX-MGE1
nEN-early1
EN-PFC3
IN-CTX-MGE2
IN-STR
EN-V1-3
EN-PFC1
IPC-nEN3
IPC-nEN1
IPC-nEN2
OPC
nIN2
nIN3
nIN1
RG-div2
RG-div1
vRG
oRG
tRG
IPC-div2
IPC-div1
Astrocyte
MGE-RG1
MGE-RG2
RG-early
MGE-IPC1
MGE-IPC2
MGE-div
MGE-IPC3
V23
U4
nIN5
U3
nIN4
U1
U2
Endothelial
Mural
Choroid
Microglia
CYCLING PROGENITORS
TOP2A
AURKA (G2/M)
CLSPN (S)
NEURAL PROGENITORS
PAX6
SOX2
HES1
EARLY RADIAL GLIA
FGFR3
LATE RADIAL GLIA
TNC
CD9
OUTER RADIAL GLIA
HOPX
FAM107A
TRUNCATED RG
CRYAB
GABAERGIC / MIXED
DLX1
DLX2
INTERMEDIATE PROGENITORS
HES6
EOMES
NEUROG1
IMMATURE GluN
SLC17A6
TELENCEPHALON
FOXG1
CORTEX
EMX1
GLUTAMATERGIC NEURONS
TBR1
BCL11B
SLC17A7
mGluN SUBTYPES
CUX2
MATURE GLUTAMATERGIC NEURON SUBTYPES
FEZF2
SATB2
VASCULAR LEPTOMENINGEAL
LUM
DCN
CHOROID PLEXUS
FOLR1
TTR

Figure S7. Classification of cell types in single-cell RNA sequencing data. a) Cell type annotation was bioinformatically aided with the Clustifyr package by correlation of the transcriptome of each cluster identified by Seurat to a reference fetal tissue dataset published by Nowakowski *et al.,* 2017. **b)** Uniform manifold approximation and projection (UMAP) location of the corresponding clusters in **a**. **c)** Feature expression for all cells in UMAP space of additional canonical cell type markers. *TOP2A* shows actively dividing cells with *AURKA* specific to G2/M phase and *CLSPN* to S phase; *PAX6, SOX2,* and *HES1* colocalization denote neural progenitor populations; *FGFR3* marks early radial glia groups; *TNC* and *CD9* are enriched in late radial glia; *HOPX* and *FAM107A* are enriched in outer radial glia; *CRYAB* is strongly expressed in the truncated radial glia group; *DLX1* and *DLX2* expression are highest in the inhibitory neurons, but also lowly expressed in the mixed immature neuron population; IPCs are determined by the colocalization of *HES6, EOMES,* and *NEUROG1*; immature glutamatergic neurons express *SLC17A6*; glutamatergic neurons express telencephalic marker *FOXG1* and cortical transcript *EMX1* in addition to neuronal transcription factors *TBR1, BCL11B,* and glutamate transporter *SLC17A7*; mature glutamatergic neuron groups are further defined by subgroups with high expression of cortical layer II-III marker *CUX2*, subcortical projection marker *FEZF2,* or upper layer marker SATB2. Small subpopulations express late radial glia transcripts, but more highly express markers of vascular leptomeningeal cells such as *LUM* and *DCN* or choroid plexus markers such as *FOLR1* and *TTR.* Scale is in units of $\log_{10}$(value + 0.1) and extends from 0-2 for all features except *HES6* and *TTR*, which extend from 0-3. Data in all panels is shown from nine organoids across four independent differentiations (Day 40: one C1, one C3, one CRISPR R203W, one A1, and one A2 organoid; Day 88: two C2, one CRISPR R203W, and one A1 organoid. See **Table S1**).

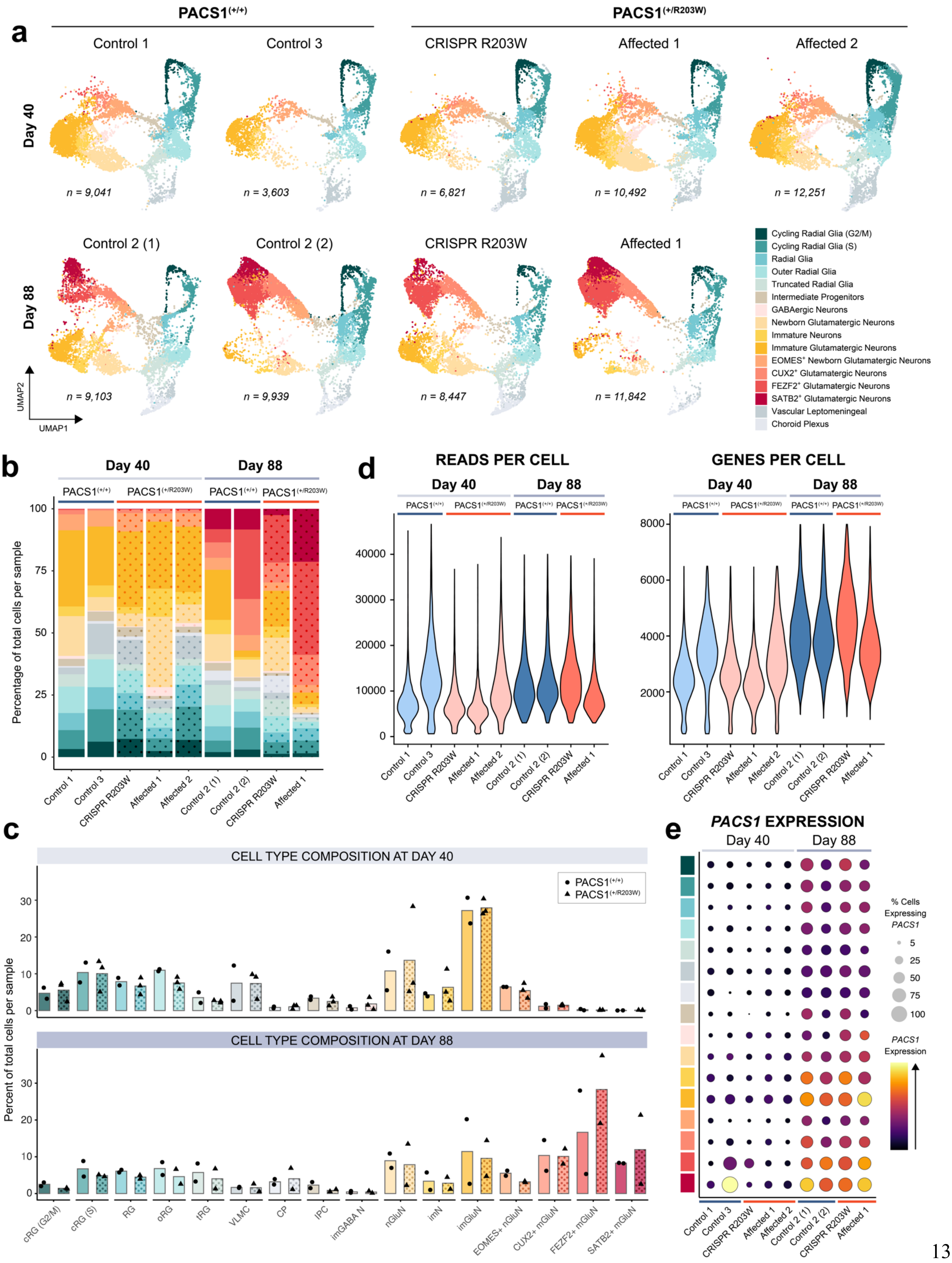
a
PACS1(+/+)
PACS1(+/R203W)
Control 1
Control 3
CRISPR R203W
Affected 1
Affected 2
Day 40
n = 9,041
n = 3,603
n = 6,821
n = 10,492
n = 12,251
Control 2 (1)
Control 2 (2)
CRISPR R203W
Affected 1
Day 88
n = 9,103
n = 9,939
n = 8,447
n = 11,842
UMAP2
UMAP1
Cycling Radial Glia (G2/M)
Cycling Radial Glia (S)
Radial Glia
Outer Radial Glia
Truncated Radial Glia
Intermediate Progenitors
GABAergic Neurons
Newborn Glutamatergic Neurons
Immature Neurons
Immature Glutamatergic Neurons
EOMES+ Newborn Glutamatergic Neurons
CUX2+ Glutamatergic Neurons
FEZF2+ Glutamatergic Neurons
SATB2+ Glutamatergic Neurons
Vascular Leptomeningeal
Choroid Plexus
b
Day 40
Day 88
PACS1(+/+)
PACS1(+/R203W)
Percentage of total cells per sample
100
75
50
25
0
Control 1
Control 3
CRISPR R203W
Affected 1
Affected 2
Control 2 (1)
Control 2 (2)
CRISPR R203W
Affected 1
d
READS PER CELL
GENES PER CELL
Day 40
Day 88
40000
30000
20000
10000
0
8000
6000
4000
2000
c
CELL TYPE COMPOSITION AT DAY 40
CELL TYPE COMPOSITION AT DAY 88
Percent of total cells per sample
PACS1(+/+)
PACS1(+/R203W)
30
20
10
0
cRG (G2/M)
cRG (S)
RG
oRG
tRG
VLMC
CP
IPC
imGABA N
nGluN
imN
imGluN
EOMES+ nGluN
CUX2+ mGluN
FEZF2+ mGluN
SATB2+ mGluN
e
PACS1 EXPRESSION
Day 40
Day 88
% Cells Expressing PACS1
5
25
50
75
100
PACS1 Expression

Figure S8. Single-cell RNA sequencing analysis reveals progression of neuronal maturity in cerebral organoids but no difference in cell type composition between genotypes. a) Uniform manifold approximation and projection (UMAP) plot of each sample colored according to cell type. Data in **a-e** is shown from nine organoids total across four independent differentiations (Day 40: one C1, one C3, one CRISPR R203W, one A1, and one A2 organoid; Day 88: two C2, one CRISPR R203W, and one A1 organoid. See **Table S1**). **b)** Bar chart of the distribution of cell type proportions between all nine samples. Data from PACS1$^{(+/R203W)}$ lines is indicated by the dot pattern. **c)** Quantification of the bar chart in **b** showing the percentage contribution of each cell type to PACS1$^{(+/+)}$ and PACS1$^{(+/R203W)}$ samples at each time point. Data from PACS1$^{(+/R203W)}$ lines is indicated by the dot pattern. $p > 0.05$ for each condition using a two sample two-sided t test. **d)** Violin plot of reads and genes captured per cell for each sample. **e)** Dot plot of *PACS1* expression for all cell types and samples. Yellow color indicates highest normalized expression and size correlates to the percentage of cells in the group that express *PACS1*.

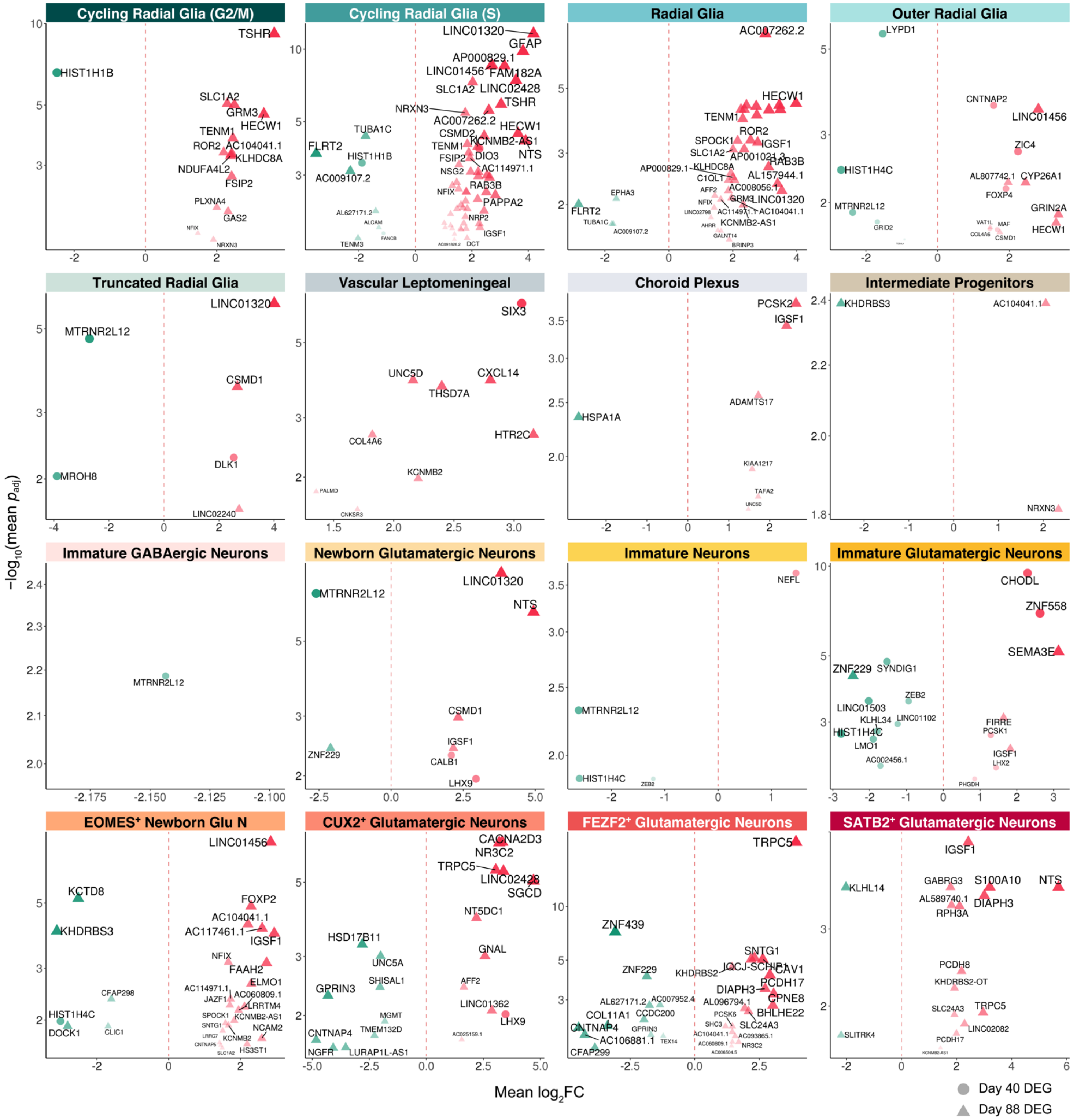

Figure S9. All differentially expressed genes. A volcano plot of the 277 significant differentially expressed genes (DEGs) by cell type. The x axis denotes mean $\log_2$FC of the DEG between standard Monocle3 and pseudobulk DESeq2 methods. The y axis denotes the mean adjusted p value of the DEG between methods using a Wald test with Benjamini-Hochberg correction. y axis is shown on a $\log_{10}$ scale with a cutoff of $p_{adj} < 0.05$ for both DEG identification methods used. Size and alpha correlates to a combined rank of lowest mean p_{adj} and highest mean absolute $\log_2$FC. Shape symbolizes the dataset time point at which the DEG was identified. Red color indicates the gene is upregulated and green downregulated in PACS1$^{(+/R203W)}$ cells. Source data can be found in the Source Data file.

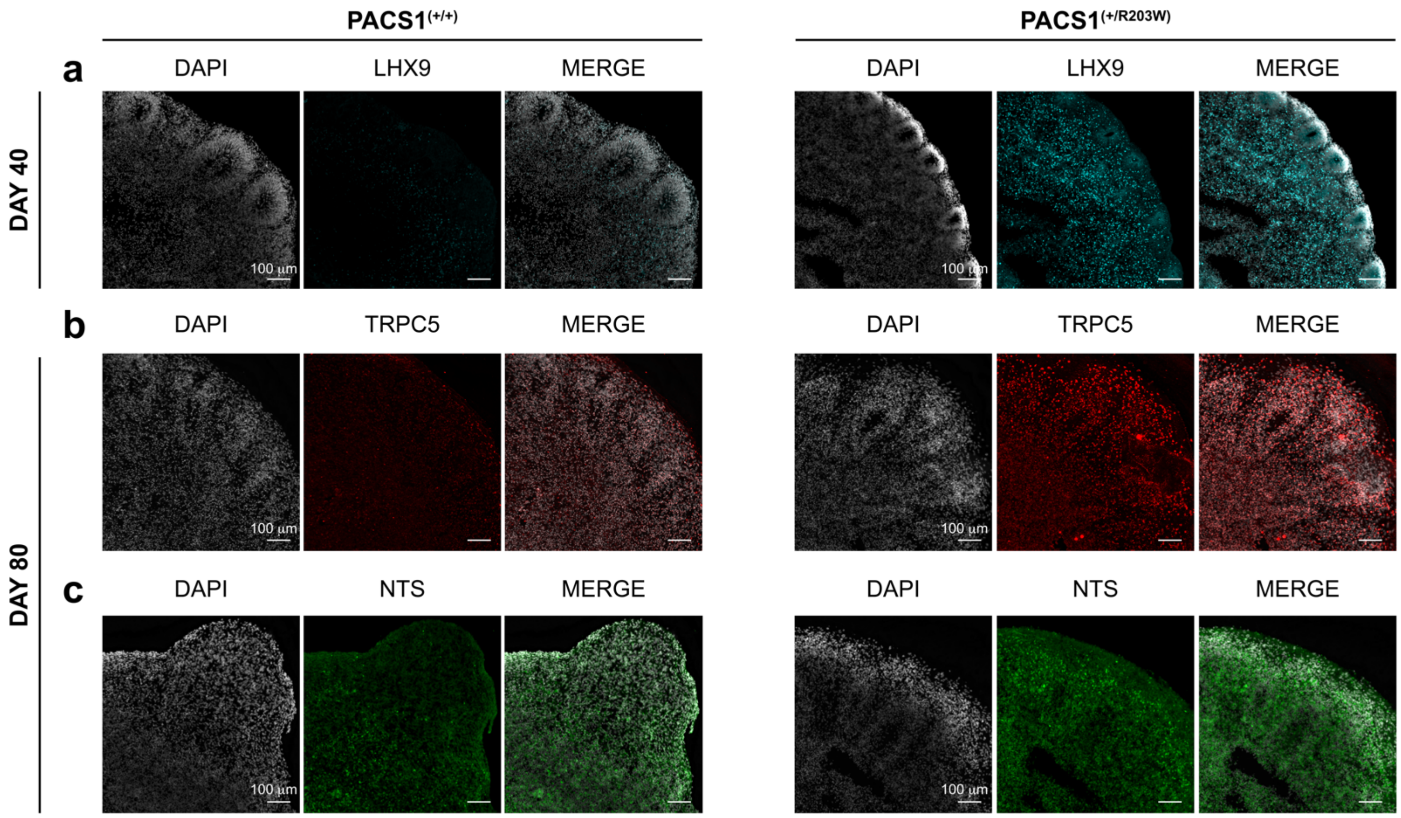

Figure S10: Protein expression of top upregulated differentially expressed genes in PACS1$^{(+/R203W)}$ organoids identified with single-cell RNA sequencing. a) Expression of LHX9 in day 40 PACS1$^{(+/+)}$ and PACS1$^{(+/R203W)}$ organoids. Scale bar = 100 µm. Single-cell RNA sequencing (scRNAseq) analysis predicted upregulation of LHX9 in PACS1$^{(+/R203W)}$ day 40 organoids in bulk, newborn glutamatergic neurons, and CUX2^{+} glutamatergic neurons. **b)** Expression of TRPC5 in day 80 PACS1$^{(+/+)}$ and PACS1$^{(+/R203W)}$ organoids. Scale bar = 100 µm. scRNAseq analysis predicted upregulation of *TRPC5* in PACS1$^{(+/R203W)}$ day 88 organoids in bulk and all three mature glutamatergic neuron groups. **c)** Expression of NTS in day 80 PACS1$^{(+/+)}$ and PACS1$^{(+/R203W)}$ organoids. Scale bar = 100 µm. scRNAseq analysis predicted upregulation of *NTS* in PACS1$^{(+/R203W)}$ day 88 organoids in bulk, newborn glutamatergic neurons, cycling radial glia (S), and SATB2^{+} glutamatergic neurons.

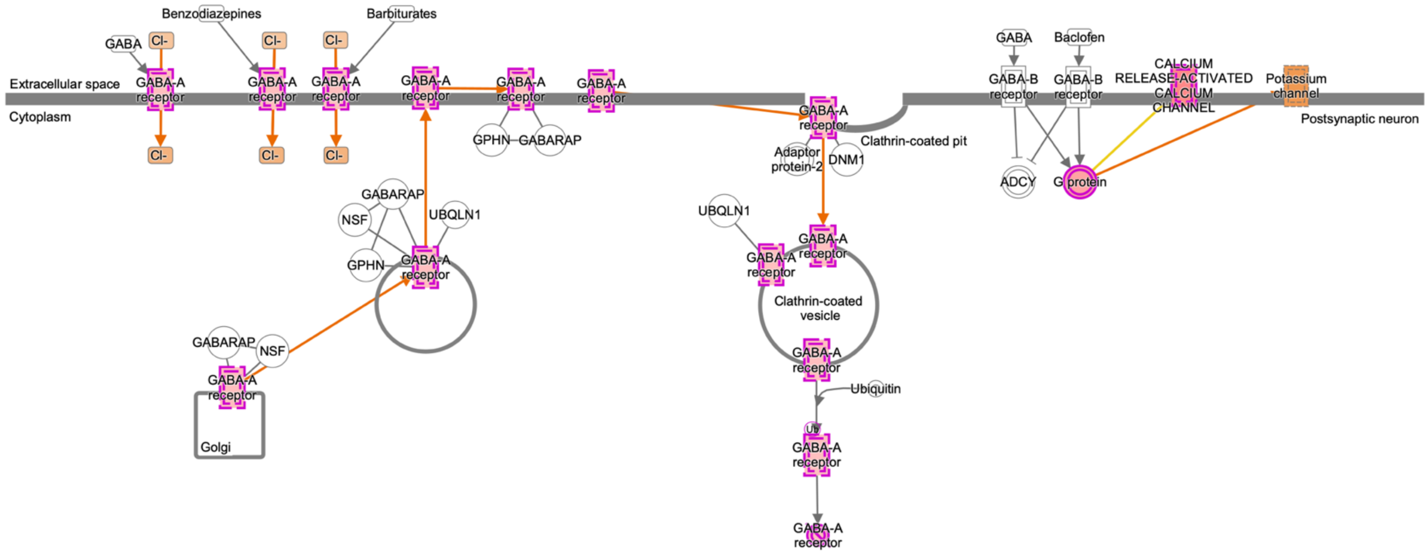

Figure S11. Ingenuity Pathway Analysis predicts differentially expressed genes in PACS1$^{(+/R203W)}$ neurons result in increased GABA-A receptor activity. a) Pathway diagram generated by Qiagen Ingenuity Pathway analysis predicting upregulation of GABA-A receptor activity in PACS1$^{(+/R203W)}$ neurons (p_{adj} = 0.016 using a right-tailed Fisher's Exact test with a Benjamini-Hochberg correction).

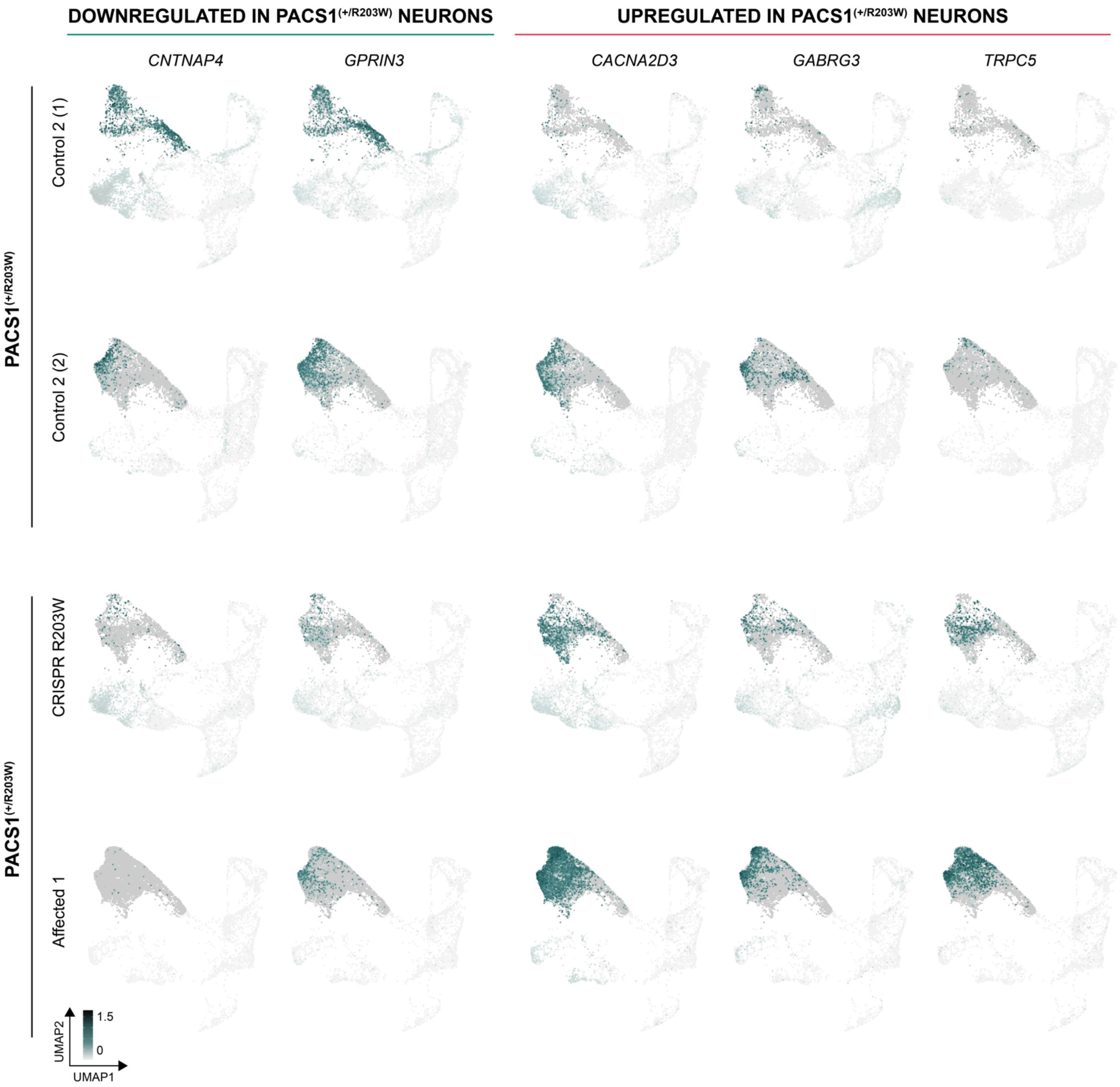

Figure S12. Visualization of top differentially expressed genes by sample demonstrates consistent patterns between PACS1$^{(+/+)}$ and PACS1$^{(+/R203W)}$ lines. Feature plots of top differentially expressed genes in PACS1$^{(+/R203W)}$ mature neurons implicated in Ingenuity Pathway analysis. Expression is shown across each day 88 organoid sequenced. Cells have been down-sampled so the same number is shown for each genotype. Scale is in units of $\log_{10}$(value + 0.1). The opacity of non-mature neuron groups has been decreased in order to highlight the implicated cell types. Data is shown from two C2, one CRISPR R203W, and one A1 day 88 organoid from one independent differentiation.

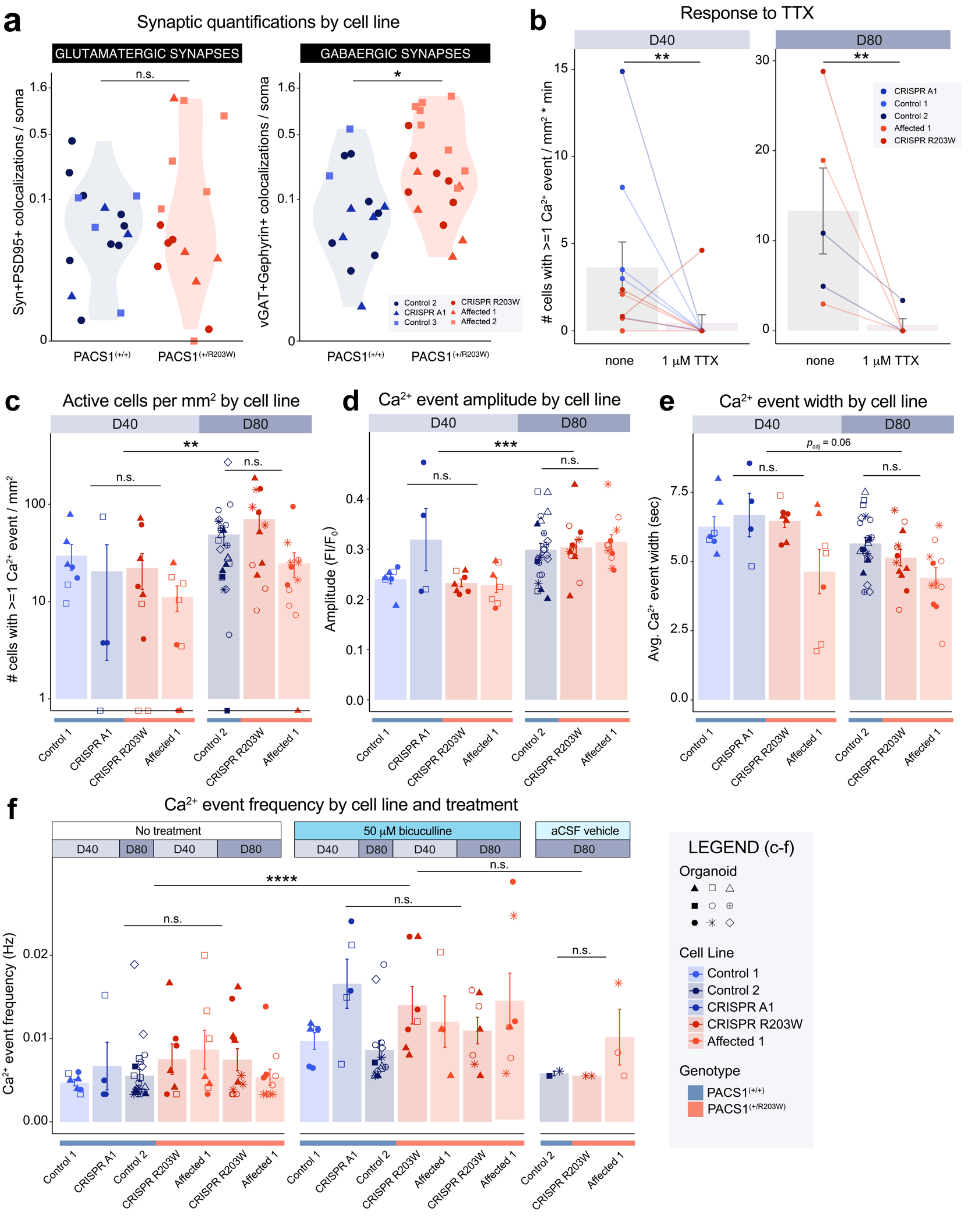

a
Synaptic quantifications by cell line
GLUTAMATERGIC SYNAPSES
GABAERGIC SYNAPSES
Syn+PSD95+ colocalizations / soma
vGAT+Gephyrin+ colocalizations / soma
n.s.
*
PACS1(+/+)
PACS1(+/R203W)
Control 2
CRISPR A1
Control 3
CRISPR R203W
Affected 1
Affected 2
b
Response to TTX
D40
D80
cells with >=1 Ca2+ event / mm2 * min
**
none
1 μM TTX
CRISPR A1
Control 1
Control 2
Affected 1
CRISPR R203W
c
Active cells per mm2 by cell line
cells with >=1 Ca2+ event / mm2
d
Ca2+ event amplitude by cell line
Amplitude (Fl/F0)
e
Ca2+ event width by cell line
Avg. Ca2+ event width (sec)
padj = 0.06
***
Control 1
CRISPR A1
CRISPR R203W
Affected 1
Control 2
f
Ca2+ event frequency by cell line and treatment
No treatment
50 μM bicuculline
aCSF vehicle
Ca2+ event frequency (Hz)
****
LEGEND (c-f)
Organoid
Cell Line
Control 1
Control 2
CRISPR A1
CRISPR R203W
Affected 1
Genotype
PACS1(+/+)
PACS1(+/R203W)

Figure S13. PACS1$^{(+/R203W)}$ organoids have increased GABAergic synaptic density but no differences in neural activity detected by calcium imaging. a) Data from **Fig. 4b-c** shown by cell line. In the left panel, dots represent number of SYN1^{+}PSD95^{+} colocalizations per soma in day 80 organoid sections. y axis is on a $\log_2$ scale. Data is from n = 16 total sections across two C2, one C3, and one CRISPR A1 PACS1$^{(+/+)}$ organoids and n = 15 total sections across two CRISPR R203W, one A1, and two A2 PACS1$^{(+/R203W)}$ organoids from three independent differentiations. See **Table S1**. U=118, p = 0.953 using a two-sided Mann-Whitney test. In the right panel, dots are number of vGAT^{+}Gephyrin^{+} colocalizations in day 80 organoid sections shown in **Fig. 4c** by cell line. y axis is on a $\log_2$ scale. Data is from n = 14 sections across two C2, one C3, and one CRISPR A1 PACS1$^{(+/+)}$ organoids and n = 20 sections across two CRISPR R203W, one A1, and two A2 PACS1$^{(+/R203W)}$ organoids from two independent differentiations. See **Table S1**. U=71, p = 0.015 calculated using a two-sided Mann-Whitney test. **b)** Number of cells with at least one Ca^{2+} event per mm^2 of an organoid section after exposure to 1 µM tetrodotoxin (TTX) per minute of recording. TTX has an effect on number of active cells (W = 84, p = 0.008) using a Wilcoxon signed rank test on the difference between paired values (p_{Shapiro} = 0.012). Data is from n = 5 PACS1$^{(+/+)}$ D40 organoids (three C1, two CRISPR A1), n = 5 PACS1$^{(+/R203W)}$ D40 organoids (three A1, two CRISPR R203W), n = 2 C2 PACS1$^{(+/+)}$ D80 organoids, and n = 2 PACS1$^{(+/R203W)}$ D80 organoids (one A1, one CRISPR R203W). **c)** Results from **Fig. 4e** shown by cell line. Bar chart showing mean ± SEM of number of active cells per mm^2 of an organoid section. Scale is $\log_{10}$ for clarity. There is an effect of day ($F_{1,23}$=8.576, p_{adj} = 0.008) but not genotype ($F_{1,23}$=0.482, p_{adj} = 0.495) using a two-way ANOVA test with a Tukey HSD correction on log-transformed values. Shapes represent values recorded from distinct n = 5 Day 40 PACS1$^{(+/+)}$ organoids (three C1 and two CRISPR A1), n = 6 Day 40 PACS1$^{(+/R203W)}$ organoids (three CRISPR R203W and three A1), n = 8 Day C2 80 PACS1$^{(+/+)}$ organoids, and n = 7 Day 80 PACS1$^{(+/R203W)}$ organoids (four CRISPR R203W and three A1) from two independent differentiations for panels **c-e**. **d)** Data from **Fig. 4f** shown by cell line. Bar chart displaying the mean ± SEM of Ca^{2+} event amplitude. There is an effect of day ($F_{1,23}$=15.481, p_{adj}=6.815E-04) but not genotype ($F_{1,23}$=0.484, p_{adj}=0.494) using a two-way ANOVA test with a Tukey HSD correction. **e)** Data from **Fig. 4g** shown by cell line. Bar chart displaying the mean ± SEM of Ca^{2+} event width. There may be an effect of day ($F_{1,23}$=3.811, p_{adj}=0.064) but not genotype ($F_{1,23}$=2.648, p_{adj}=0.117) using a two-way ANOVA test with a Tukey HSD correction. **f)** Bar chart displaying mean ± SEM of average frequency of Ca^{2+} events for each cell line in **Fig. 4h**, as well as any non-paired values. There is an effect of 50 µM bicuculline (BCU) exposure on Ca^{2+} event frequency (H_1= 18.468, p_{adj} = 8.6E-05) but no other parameters tested using one-sided Kruskal Wallis tests followed by Benjamini Hochberg correction (p_{Shapiro} = 6.6E-04). Shapes represent values recorded from distinct n = 5 Day 40 PACS1$^{(+/+)}$ organoids (three C1 and two CRISPR A1), n = 6 Day 40 PACS1$^{(+/R203W)}$ organoids (three CRISPR R203W and three A1), n = 8 Day C2 80 PACS1$^{(+/+)}$ organoids, and n = 8 Day 80 PACS1$^{(+/R203W)}$ organoids (four CRISPR R203W and four A1) from two independent differentiations. Legend also applies to panels c-e. All data can be found in the Source Data file.

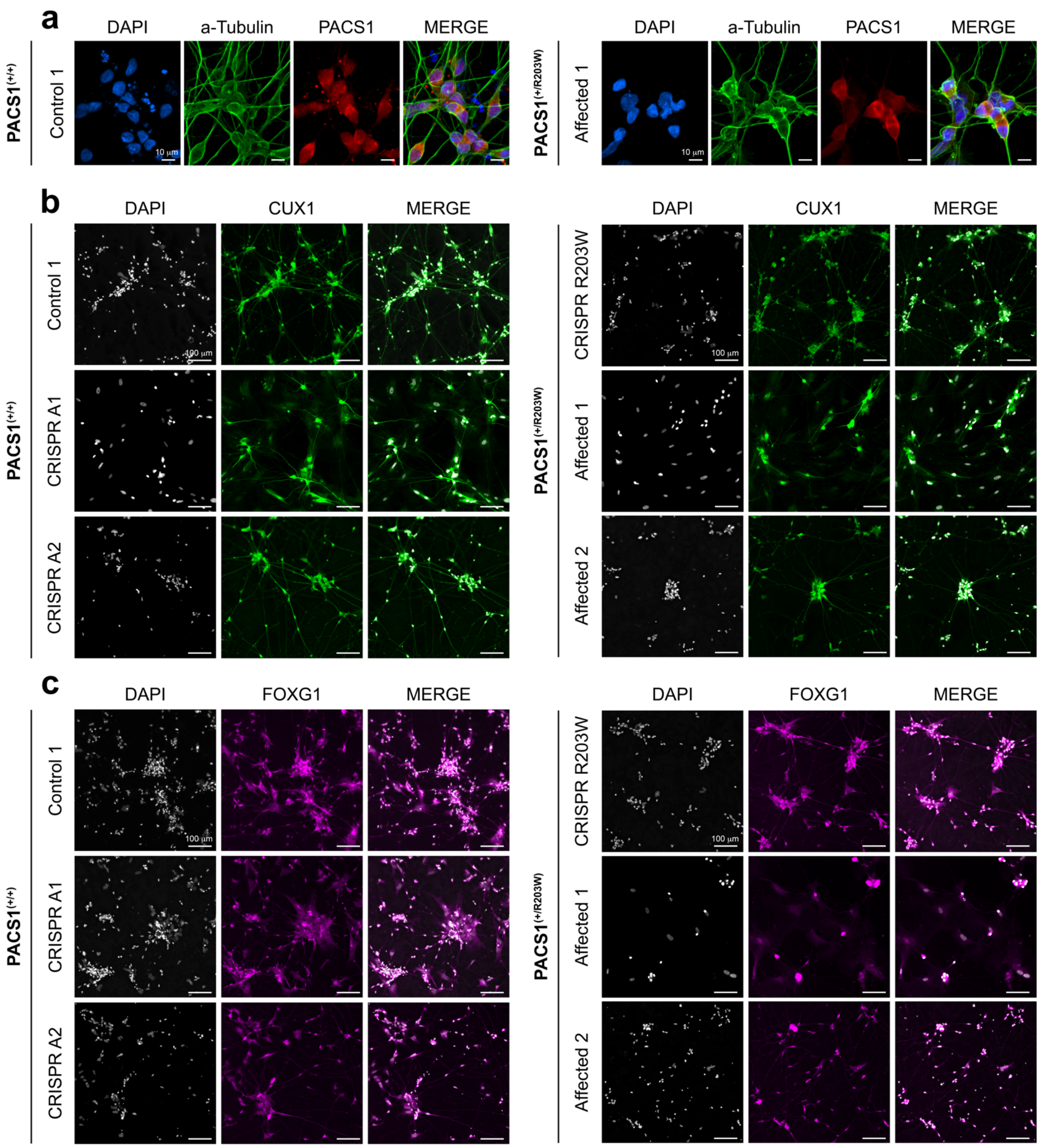

Figure S14. NEUROG2-derived neurons generated from iPSCs express forebrain markers and PACS1. a) Day 14 C1 PACS1$^{(+/+)}$ neurons and A1 PACS1$^{(+/R203W)}$ neurons express PACS1. Scale bar = 10 µm. **b)** Both PACS1$^{(+/+)}$ and PACS1$^{(+/R203W)}$ ~D50 NEUROG2 neurons express layer II/III neuron marker CUX1 and the telencephalic transcription factor FOXG1 in **c)**. Scale bar = 100 µm.

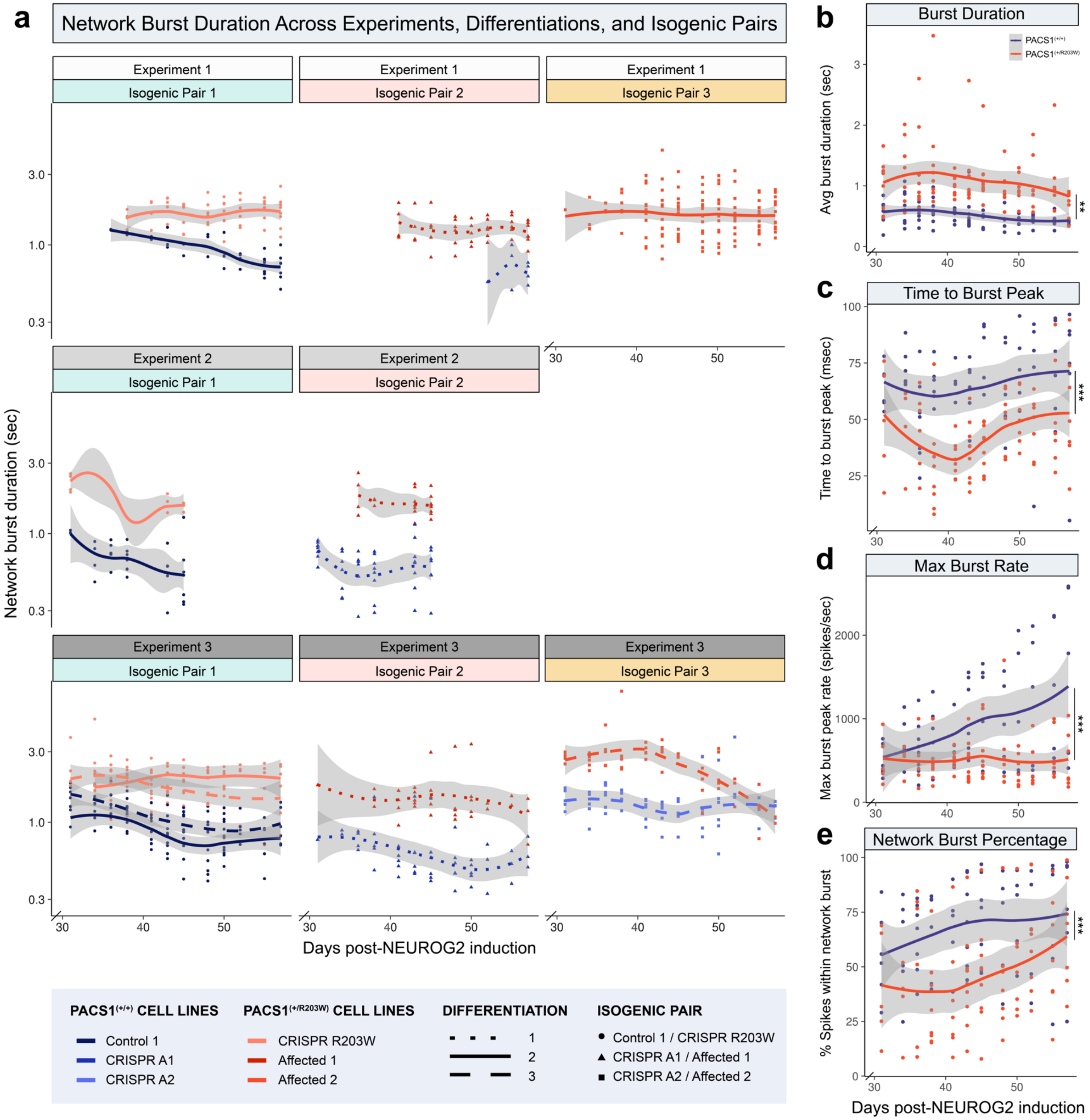

Figure S15. PACS1$^{(+/R203W)}$ neurons consistently display a prolonged network burst duration. a) Dots show multielectrode array (MEA) well averages of network burst duration for each experiment, differentiation, and isogenic pair. The x axis has been abbreviated to days 30-57 as network bursts do not occur before day 30. Dots represent biological replicate averages from MEA wells. The smoothed line represents genotype averages with the grey band indicating 95% confidence interval. n = 8 PACS1$^{(+/+)}$ lines (four C1, three CRISPR A1, and one CRISPR A2) and n = 9 PACS1$^{(+/R203W)}$ lines (four CRISPR R203W, three A1, and two A2) from three independent experiments. See **Table S1**. Missing values are either due to either: the neurons not yet being mature enough to elicit network bursting activity; the line not being included in the MEA experiment; or omission of the data upon observation of excessive clumping or other indicators of poor viability. **b-e)** Further

MEA metrics for neurons days 30-57 of differentiation. Dots represent averages from each cell line, while the smoothed curve shows genotype averages with error bands indicating a 95% confidence interval from n = 8 PACS1$^{(+/+)}$ lines (four C1, three CRISPR A1, and one CRISPR A2) and n = 9 PACS1$^{(+/R203W)}$ lines (four CRISPR R203W, three A1, and two A2) from three independent experiments. p values were calculated by conducting an ANOVA between a linear regression with and without genotype included as a covariate. In instances where p values are reported between specific time points, a Kruskal Wallis test was run. **b)** Average burst duration in seconds. Genotype had a significant effect on burst duration ($F_{0,2}$ = 5.261, p = 0.006). **c)** Time from the start to the peak of the average network burst. Genotype had a significant effect on time to burst peak ($F_{0,2}$ = 26.686, p = 8.48E-11). **d)** Maximum number of spikes per second in the average network burst. Genotype had a significant effect on max burst rate ($F_{0,2}$ = 27.413, p = 4.89E-11). **e)** Percentage of total spikes that occur within network bursts. Genotype had a significant effect on network burst percentage ($F_{0,2}$ = 14.705, p = 1.30E-06). Individual time points are significant at days 38 – 43 of differentiation. All data can be found in the Source Data file.

SUPPLEMENTARY TABLES

	PACS1(+/+)					PACS1(+/R203W)					
Cell line	Control 1	Control 2	Control 3	CRISPR A1	CRISPR A2	CRISPR R203W	Affected 1	Affected 2			
Identifaction/Line of Origin	GM03651	GM03652	GM27160	GM27161	GM27159	GM03651	GM27161	GM27159	Value represents	Independent Differentiations	Applicable Figures
Sex	F	M	F	M	F	F	M	F			
NPC BRDU assay	1	1	1	0	0	1	1	1	Line diff avg / experiment	2	Fig S5c-d
NPC TUNEL assay	2	0	1	0	0	3	1	2	Line diff avg / experiment	3	Fig S5e-f
FOXG1/TBR1/SATB2 D40 org	3	0	0	0	0	1	2	0	One organoid	4+	Fig 1d, S4d
FOXG1/TBR1/SATB2 D80 org	0	2	0	1	0	2	1	0	One organoid	3+	Fig 1d, S4d
CTIP2/SOX2 D40 org	5	0	1	1	1	1	5	1	One organoid	4+	Fig 1e, S4e
CTIP2/SOX2 D80 org	2	3	1	1	0	3	4	0	One organoid	3+	Fig 1e, S4e
PACS1 western blot D40 org	2	3	0	2	0	4	4	0	One organoid	2	Fig 1f-g
scRNAseq day 40 org	1	0	1	0	0	1	1	1*	One organoid	3	Figs 2-3, S6-S9
scRNAseq day 88 org	0	2	0	0	0	1	1	0	One organoid	1	Figs 2-3, S6-S9
Syn1/PSD95 D79 org	0	2	1	1	0	2	1	2	One organoid	3	Fig 4b, S13a
vGAT/GPHN D79 org	0	2	1	1	0	2	1	2	One organoid	2+	Fig 4c, S13a
Calcium imaging D40 org	3	0	0	2	0	3	3	0	One organoid	1	Fig 4d-i, S13b-f
Calcium imaging D80 org	0	8	0	0	0	4	4	0	One organoid	1	Fig 4d-i, S13b-f
Sholl NEUROG2 neurons	1	0	0	2	1	1	2	1	Line diff avg / experiment	2	Fig 5d-g
MEAs NEUROG2 neurons	4	0	0	3	1	4	3	2	Line diff avg / experiment	3	Fig 5i-o, S15

Table S1. Number of samples from each cell line contributing to each experiment.
Distribution of samples from each cell line used for each assay. * Indicates a specific instance in which two technical replicates were combined and treated as one organoid. + indicates at least that many differentiations were used in quantifications. Line diff avg / experiment means each cell line per differentiation per experiment is considered a biological replicate. Please note that independent differentiations indicates the total number of differentiations present across all samples in a given experiment, but does not necessarily mean the experiment was repeated that many times with the exact same lines.

Data S1. The complete list of differentially expressed genes. (Separate file)
The complete list of differentially expressed genes (DEGs) between PACS1$^{(+/+)}$ and PACS1$^{(+/R203W)}$ organoids identified with single-cell RNA sequencing.
