# Supplementary material for: iPSC-derived models of PACS1 syndrome reveal transcriptional and functional deficits in neuron activity
Source: Nat Commun. 2024 Jan 27;15:827. doi: 10.1038/s41467-024-44989-7 (PMC10821916; doi:10.1038/s41467-024-44989-7)
Supplement: Supplementary file 3 — Description of Additional Supplementary Files [file 41467_2024_44989_MOESM3_ESM.pdf]

Description of Additional Supplementary Files

File Name: Data S1
Description: **The complete list of differentially expressed genes.** The complete list of differentially expressed genes (DEGs) between PACS1$^{(+/+)}$ and PACS1$^{(+/R203W)}$ organoids identified with single-cell RNA sequencing.
