# Supplementary material for: iPSC-derived models of PACS1 syndrome reveal transcriptional and functional deficits in neuron activity
Source: Nat Commun. 2024 Jan 27;15:827. doi: 10.1038/s41467-024-44989-7 (PMC10821916; doi:10.1038/s41467-024-44989-7)
Supplement: Supplementary file 5 — Reporting Summary [file 41467_2024_44989_MOESM5_ESM.pdf]

Corresponding author(s): Alicia Guemez-Gamboa
Last updated by author(s): December 18th, 2023

Reporting Summary

Nature Portfolio wishes to improve the reproducibility of the work that we publish. This form provides structure for consistency and transparency in reporting. For further information on Nature Portfolio policies, see our Editorial Policies and the Editorial Policy Checklist.

Statistics

For all statistical analyses, confirm that the following items are present in the figure legend, table legend, main text, or Methods section.

n/a	Confirmed	
☐	☒	The exact sample size (*n*) for each experimental group/condition, given as a discrete number and unit of measurement
☐	☒	A statement on whether measurements were taken from distinct samples or whether the same sample was measured repeatedly
☐	☒	The statistical test(s) used AND whether they are one- or two-sided<br>*Only common tests should be described solely by name; describe more complex techniques in the Methods section.*
☐	☒	A description of all covariates tested
☐	☒	A description of any assumptions or corrections, such as tests of normality and adjustment for multiple comparisons
☐	☒	A full description of the statistical parameters including central tendency (e.g. means) or other basic estimates (e.g. regression coefficient) AND variation (e.g. standard deviation) or associated estimates of uncertainty (e.g. confidence intervals)
☐	☒	For null hypothesis testing, the test statistic (e.g. *F*, *t*, *r*) with confidence intervals, effect sizes, degrees of freedom and *P* value noted<br>*Give P values as exact values whenever suitable.*
☒	☐	For Bayesian analysis, information on the choice of priors and Markov chain Monte Carlo settings
☐	☒	For hierarchical and complex designs, identification of the appropriate level for tests and full reporting of outcomes
☐	☒	Estimates of effect sizes (e.g. Cohen's *d*, Pearson's *r*), indicating how they were calculated

Our web collection on statistics for biologists contains articles on many of the points above.

Software and code

Policy information about availability of computer code

Data collection

Images for immunohistochemistry were captured on a Nikon W1 confocal microscope at Northwestern's Center for Advanced Microscopy & Nikon Imaging Center. Output files were analyzed using ImageJ (Fiji v2.3.0) and CellProfiler (v4.2.4) and statistics were calculated in R (v4.0.3-v4.1.1).

Western blot scans in Fig. 1f were captured using the Odyssey Fc Imaging System (Li-Cor) and quantified with Image Studio (Li-Cor). Statistics on protein abundance ratios were done using GraphPad Prism (v8.4.2).

For single-cell RNA sequencing, Day 40 organoid samples were sequenced on an Illumina HiSeq 4000 and day 88 samples were sequenced on an Illumina NovaSeq 6000 due to changes in Northwestern's scRNAseq pipeline. This is reflected in the average number of reads and genes captured per cell between timepoints (Fig. S8d), so differential expression analysis was performed separately. Raw data was demultiplexed and reads were aligned to GRCh38 at Northwestern's Center for Genetic Medicine using the CellRanger pipeline (v3.0.2 for C1, C3, CRISPR-R203W, and A1 day 40 samples; v4.0.0 for the A2 day 40 sample, and v7.0.1 for all day 88 samples due to the number years spanning this project). Output matrix files were analyzed in R (v4.0.3-v4.1.1) using packages including Seurat (v4.0.0), Monocle3 (v1.0.0), DoubletFinder (v2.0.3), DoubletDecon (v1.1.6), Clustifyr (v1.2.0), DESeq2 (v1.30.1), topGO (v2.42.0), rrvgo (v1.9.1), and webr (v0.1.5). The Qiagen Ingenuity Pathway Analysis tool (v1.21.03) was also used to analyze pathway enrichments in differentially expressed genes.

Calcium imaging recording was performed on a Nikon W1 confocal microscope. Sections were imaged in a live-cell chamber at 37°C 5% CO_2 with a Nikon W1 spinning disk confocal microscope for 1-5 minutes (depending on the condition) at 2 Hz. Calcium signal was extracted in Fiji (v2.3.0) by calculating average intensity for each frame over regions of interest with the greatest Z score. Traces were processed in Matlab (vR2022a) using code developed by Marc Dos Santos, which is available at https://github.com/marcdossantosPHD/PenzesLabimaging. In brief, data were normalized to a rolling median background of 25 seconds. Peaks were detected using the findpeaks function (MinPeakDistance = 3

seconds, MinPeakHeight = Δ0.17 F/F0, MinPeakProminence Δ0.10 F/F0, MaxPeakWidth = 9 seconds). Cells with no detected peaks were removed from the analysis, except for paired TTX recordings. Output metrics were analyzed in R (v4.0.3-v4.1.1).

Sholl analysis images were captured using a BZX710 Keyence epifluorescent microscope. Images were analyzed using the Sholl plugin on Fiji (v4.0.1).

Spontaneous and stimulated neuronal activity was recorded using Axion Biosystems Maestro 768 channel amplifier and Axion Integrated Studios (AxIS) v2.5 software. The amplifier recorded from all channels simultaneously using a gain of 1200x and a sampling rate of 12.5 kHz/channel. After passing the signal through a Butterworth band-pass filter (300–5000 Hz), on-line spike detection (threshold = 6 x the root-mean-square of noise on each channel) was performed with the AxIS adaptive spike detector. All recordings were conducted at 37°C in 5% CO_2/95% O_2. Spontaneous network activity was recorded for 5 min each day starting on day 10 of differentiation. Starting day 10, neurons were also electrically stimulated with 20 pulses at 0.5 and 0.25 Hz after spontaneous recordings were made to facilitate maturation and migration of neurons to the electrode field. After each recording session, cells were observed under a microscope to check for excessive clumping or other indicators of suboptimal recording conditions. Data was omitted if cells did not look healthy and distributed enough across the electrode field to produce high-quality recordings. Spike files were processed with the Axis Neural Metric Tool (v3.1.7). Electrode burst settings were algorithm = Poisson surprise, min surprise = 5. Network burst settings were algorithm = ISI threshold, min # spikes = 50, max ISI (ms) = 100, min % of electrodes = 18, synchrony window = 20. Average network burst settings were network burst window start = 0 ms, network burst window end = 100 ms, bin size = 1 ms. Metrics generated from these output parameters were further investigated in R (v4.0.3-v4.1.1).

Data analysis

All code is available upon request. Code used in this study is a compilation from the following sources. Reads were aligned by Northwestern University's Center for Genetic Medicine using the 10X Genomics Cell Ranger pipeline found at https://support.10xgenomics.com/single-cell-gene-expression/software/pipelines/latest/using/tutorial_ov. The output matrix files were processed with Seurat and Monocle3 by following vignettes developed by the Satija lab (https://github.com/satijalab/seurat) and Trapnell lab (https://github.com/cole-trapnell-lab/monocle3) respectively. Differentially expressed genes were determined by consensus results from Monocle3 approaches and a DESeq2 workflow specifically developed for pseudobulk single-cell analysis (https://hbctraining.github.io/scRNA-seq/lessons/pseudobulk_DESeq2_scrnaseq.html). Code for calcium imaging analysis was developed by Marc Dos Santos and adapted with permission by Lauren Rylaarsdam. Examples of this pipeline are available at https://github.com/marcdossantosPHD/PenzesLabimaging. R code for analyzing multielectrode array metrics calculated with the Axis Neural Metric Tool is available upon request.

For manuscripts utilizing custom algorithms or software that are central to the research but not yet described in published literature, software must be made available to editors and reviewers. We strongly encourage code deposition in a community repository (e.g. GitHub). See the Nature Portfolio guidelines for submitting code & software for further information.

Data

Policy information about availability of data

All manuscripts must include a data availability statement. This statement should provide the following information, where applicable:

- Accession codes, unique identifiers, or web links for publicly available datasets
- A description of any restrictions on data availability
- For clinical datasets or third party data, please ensure that the statement adheres to our policy

The FASTQ and CellRanger matrix files for the single-cell RNA sequencing (scRNAseq) data generated in this study have been deposited in the NCBI Gene Expression Omnibus (GEO) database under accession code GSE250386. scRNAseq reads were aligned to the publicly available human genome reference build GRCh38. The counts for organoid immunohistochemistry analysis (Fig. 1b-e), quantifications for PACS1 abundance (Fig. 1f-g), differentially expressed genes identified with scRNAseq (Fig. 3), Sholl analysis results (Fig. 5d-g), and key metrics for calcium imaging (Fig. 4) and multielectrode array recordings (Fig. 5) generated in this study are provided in Source Data file.

Human research participants

Policy information about studies involving human research participants and Sex and Gender in Research.

Reporting on sex and gender	NA
Population characteristics	NA
Recruitment	NA
Ethics oversight	NA

Note that full information on the approval of the study protocol must also be provided in the manuscript.

Field-specific reporting

Please select the one below that is the best fit for your research. If you are not sure, read the appropriate sections before making your selection.

☒ Life sciences ☐ Behavioural & social sciences ☐ Ecological, evolutionary & environmental sciences

For a reference copy of the document with all sections, see nature.com/documents/nr-reporting-summary-flat.pdf

Life sciences study design

All studies must disclose on these points even when the disclosure is negative.

Sample size	Nine organoid samples across four batches were used for single-cell RNA sequencing. 81,539 high-quality cells were sequenced in total. This was in line with practices in the field when we began this research in 2019. For example, Birey et al. (PMC5805137) report sequencing 11,838 cells, Kanton et al. (PMID 31619793) sequenced 43,498 cells; and Trujillo et al. (PMC6778040) sequenced 15,990 cells. For other analyses, we aimed to perform experiments at least 2-3 times with multiple PACS1(+/+) and PACS1(+/R203W) lines per experiment. Power analysis was not done prior.
Data exclusions	As is necessary in single-cell sequencing data analysis, cells were filtered extensively based on metrics such as percentage of mitochondrial reads, number of genes captured, and number of total counts. One organoid sample was eventually excluded altogether from single-cell RNA sequencing analysis due to much lower cell yield than expected and poor data quality. One extreme outlier in the glutamatergic synaptic quantification assay that was 2.2 standard deviations from the next highest value and 4.2 standard deviations from the mean was excluded. Further details are described in the methods section.
Replication	Samples were submitted for single-cell RNA sequencing across four different batches. Immunohistochemistry analysis, Western blot analysis, calcium imaging, sholl analysis, and multielectrode array recordings (MEA) were performed with samples from multiple independent differentiations. Please see Table S1 for a breakdown of number of samples and independent differentiations contributing to each experiment. The neural precursor cell TUNEL assay and PACS1 protein quantification in day 40 organoids were repeated twice with the same results. Organoid quantifications in Fig. 1d-e were performed from 3-4 independent differentiations with clear differences between timepoints, but not genotype. Synaptic quantifications in Fig. 4b-c were performed with organoids from 2-3 independent differentiations. GABAergic synaptic density was consistently increased in the A2 line, but results with A1 and CRISPR A1 varied. Calcium imaging was repeated - though at different organoid timepoints - with the same conclusions reached between genotypes. Sholl analysis was repeated with no difference detected between genotypes. MEA experiments were done three independent times with PACS1(+/R203W) neurons displaying an increased interspike interval in each experiment and isogenic pair (Fig. S15a).
Randomization	Immunohistochemistry imaging and quantifications for Fig. 1d-e, Fig. 4b-c, and Fig. 5h were done blinded and randomized according to blinded names. This was typically accomplished by acquiring a list of names in a category - e.g., animals - using an online list randomizing tool, then renaming the files accordingly. The order of cell line data collection for calcium imaging was scrambled for each day of recording. The order of cell lines plated on multi-electrode arrays was rearranged for each plate.
Blinding	Organoid culturing and single-cell RNA sequencing analysis were not performed blinded. Sample genotype was necessary information in order to construct differential expression designs. The same filtering methods were applied to each genotype. Immunohistochemistry imaging and quantifications for organoid pre/postmitotic marker ratios (Fig. 1d-e), synaptic quantifications (Fig. 4b-c), and counting of synapsin+ cells on MEA wells (Fig. 5h) were done blinded. Calcium imaging and MEA recordings were not done blinded due to the automated nature of the analysis. The same parameters were applied to both genotypes.

Reporting for specific materials, systems and methods

We require information from authors about some types of materials, experimental systems and methods used in many studies. Here, indicate whether each material, system or method listed is relevant to your study. If you are not sure if a list item applies to your research, read the appropriate section before selecting a response.

Materials & experimental systems

n/a	Involved in the study	
☐	☒	Antibodies
☐	☒	Eukaryotic cell lines
☒	☐	Palaeontology and archaeology
☒	☐	Animals and other organisms
☒	☐	Clinical data
☒	☐	Dual use research of concern

Methods

n/a	Involved in the study	
☒	☐	ChIP-seq
☒	☐	Flow cytometry
☒	☐	MRI-based neuroimaging

Antibodies

Antibodies used	Primary antibodies used in this study for immunohistochemistry are: a-Tubulin (Abcam, ab7291, Clone DM1A, Lot GR3341361-10, Dilution 1:200); Brachyury (Invitrogen, 14-9770-82, Clone X1A02, Lot 2681461, Dilution 1:500); BrdU (BD Biosciences, 347580, Clone B44, Lot 6042756, Dilution 1:100); CTIP2 (Abcam, ab18465, Clone 25B6, Lot GR3420263-2, Dilution 1:200); CUX1 (Proteintech, 11733-1-AP); CXCR4 (Abcam, ab181020, Clone EPUMBR3, Lot 1002835-10, Dilution 1:200); EMX1 (Atlas Antibodies, HPA006421, Lot C106561, Dilution 1:100); FOXG1 (Abcam, ab196868, Clone EPR18987, Lot GR3242662-19, Dilution 1:100); Gephyrin (Synaptic Systems, 147-021, Clone mAb7a, Dilution 1:100); GFAP (Synaptic Systems, 173044, Dilution 1:200); GFP (Abcam, ab13970, Lot GPR236651-4, Dilution 1:200); Ki67 (Abcam, ab15580, Dilution 1:200); LHX9 (Sigma, HPA009695, Lot 000033857, Dilution 1:200); MAP2 (Invitrogen, MAB3418, Clone AP20, Dilution 1:200); Nestin (EMD Millipore, MAB5326, Lot 3430617, Dilution 1:300); NTS (Synaptic Systems, 418005, Dilution 1:500); OCT4A (Cell Signaling Technology, C30A3); OCT3/4 (R&D Systems, MAB1759, Clone 240408, Lot KQX0420031, Dilution 1:100); PACS1 (Generated by Gary Thomas's Lab; Dilution 1:200); PAX6 (BioLegend, 901301, Lot B386304, Dilution 1:100); PSD95 (NeuroMab, 75-028, Clone K28/43, Dilution 1:1000. Gift from the lab of Peter Penzes); SATB2

(Synaptic Systems, 327004, Dilution 1:100); SOX2 (EMD Millipore, AB5603, Lot 3587118, Dilution 1:200); SOX17 (Invitrogen, MA5-24885, Clone OTI3B10, Lot YE3927121, Dilution 1:100); SYN1 (Cell Signaling Technology, 5297, Clone D12G5, Dilution 1:300. Gift from the lab of Peter Penzes); TBR1 (Proteintech, 66564-1-Ig, Dilution 1:200); TRA1-60 (R&D systems, MAB1658, Clone 222328, Lot JKW0219121, Dilution 1:100); TRA1-60 (Sigma, MAB4360); TRPC5 (Invitrogen, MA5-27657, Clone N67/15, Lot YE3931332, Dilution 1:500); and vGAT (Thermo Scientific, PA5-27569, Lot WL3447439, Dilution 1:200).

Secondary antibodies used in this study include: donkey anti-mouse Alexa Fluor 488 (Invitrogen, A21202, Lot 1890861); donkey anti-mouse Alexa Fluor 594 (Invitrogen, A21203, Lot 2294985); donkey anti-mouse Alexa Fluor 647 (Invitrogen, A31571, Lot 2720365); donkey anti-rabbit Alexa Fluor 488 (Invitrogen, A21206, Lot 1874771); donkey anti-rabbit Alexa Fluor 594 (Invitrogen, A21207, Lot 2441375); donkey anti-rabbit Alexa Fluor 647 (Invitrogen, A31573, Lot 2359136); donkey anti-chicken Alexa Fluor 488 (Jackson ImmunoResearch, 703-545-155, Lot 119854); donkey anti-chicken Alexa Fluor 594 (Jackson ImmunoResearch, 703-585-155, Lot 147524); donkey anti-rat Alexa Fluor 488 (Invitrogen, A21208, Lot 2273677); donkey anti-rat Alexa Fluor 594 (Invitrogen, A21209, Lot 2078918); and goat anti-guinea pig Alexa Fluor 488 (Invitrogen, A11073, Lot 46214A).

Validation

Only antibodies with references and validation from reputable sources were used. All antibodies have been validated for ICC, IHC, or IF in human tissue. One exception is SATB2 (Synaptic Systems 237004) which was validated in mouse tissue. Additional validation statements from manufacturers are listed here. Abcam antibody specificity (a-Tubulin, CTIP2, CXCR4, FOXG1, GFP, Ki67) "is confirmed by looking at cells that either do or do not express the target protein within the same tissue. Initially, our scientists will review the available literature to determine the best cell lines and tissues to use for validation. We then check the protein expression by IHC/ICC to see if it has the expected cellular localization. If the localization of the signal is as expected, this antibody will pass and is considered suitable for use in IHC/ICC." The Brachyury antibody (Invitrogen 14-9770-82) "has been tested by immunohistochemistry on formalin-fixed paraffin embedded human tissue using low pH antigen retrieval and can be used at less than or equal to 5 µg/mL. This X1AO2 antibody has also been tested by immunocytochemistry of fixed and permeabilized human cells and can be used at less than or equal to 5 µg/mL." BrdU (BD 347580) was tested using flow cytometry. CUX1 (Proteintech 11733-1-AP) and GFAP (Synaptic Systems, 173044) have been KD/KO validated. EMX1, LHX9, and PACS1 are Prestige antibodies, which "are tested by IHC tissue array of 44 normal human tissues and 20 of the most common cancer type tissues." MAP2 (Invitrogen MAB3418) is "routinely evaluated by Western Blot on Rat Brain lysate." Nestin (EMD Millipore MAB5326) was "evaluated by western blot on Huvec cell lysate." OCT3/4 (R&D Systems MAB1759) was "validated for immunocytochemistry on immersion fixed NTera-2 human testicular embryonic carcinoma cell line, D3 mouse." PAX6 (Biolegend 901301) was "tested for purity by SDS-PAGE gel electrophoresis. IgG antibodies are required to have purity >95%. Fluorophore and enzyme-conjugated antibodies follow strict manufacturing specifications to ensure performance. Each lot is validated by QC testing as stated on the TDS to confirm specificity and lot-to-lot consistency." SOX17 and vGAT (Invitrogen MA5-24885 and PA5-27569) were "verified by relative expression to ensure that the antibody binds to the antigen stated."

Eukaryotic cell lines

Policy information about cell lines and Sex and Gender in Research

Cell line source(s)

This research was performed in compliance with relevant ethical regulations and approved by the Northwestern University Institutional Review Board (IRB STU00215054). Five PACS1(+/+) and three PACS1(+/R203W) induced pluripotent stem cell (iPSC) lines were utilized in total. All donors consented to tissue being used for research purposes. Two control lines derived from a 25-year-old healthy female (C1; GM03651) and 24-year-old male (C2; GM03652) were purchased from as fibroblasts from Coriell and reprogrammed to pluripotency by the Northwestern University Stem Cell Core using Sendai viral vectors containing OCT4, SOX2, KLF4 and CMYC. A third control line was derived from an 39-year-old unaffected mother (C3; S033751*B/GM27160) of a 3-year-old daughter with PACS1 syndrome (A2; S033745*B/GM27159). These lines were purchased as iPSCs from Coriell but are now available at Wicell (PACS1001i-GM27160 and PACS1002i-GM27159). An additional 6-year-old male patient line (A1; PACS1003i-GM27161) was purchased as iPSCs from WiCell. Three isogenic pairs were generated in total using CRISPR/Cas9 gene editing technology by introducing the heterozygous variant which leads to p.R203W into the C1 background (CRISPR R203W) and correcting the variant in both patient lines (CRISPR A1 and CRISPR A2; Fig. S1).

Authentication

Karyotype of all lines was done by WiCell and determined normal (Fig. S2). Pluripotency tests of iPSC lines using the STEMdiff Trilineage Differentiation Kit (Stem Cell Technologies, 05230) according to manufacturer instructions demonstrated differentiation capacity of all three germ layers (Fig. S3). All cell lines were regularly tested for mycoplasma contamination and genotyped with Sanger sequencing. To check for off-target effects of CRISPR-generated lines and confirm the genotype, DNA from clones of interest was sent to Novogene for whole genome or whole exome sequencing. The resulting sequences were mapped using a Burrows-Wheeler Aligner (BWA, v0.7.15) and manually checked for off-target effects with the Integrative Genomics Viewer (IGV, v2.8.10). Of the top 100 off-target effects predicted by the IDT CRISPR design tool, only two other sites were exonic (MVP chr16:-29844665; PACS2 chr14:-105355130) and did not have any mismatched base pairs in any of the three isogenic sets of lines. The top five off-target sites predicted by CHOPCHOP (v3) were all non-coding, but analysis of the CRISPR R203W and CRISPR A1 lines - which received whole genome sequencing analysis - did not reveal any unintended edits.

Mycoplasma contamination

All cell lines were tested multiple times throughout the course of these experiments for mycoplasma contamination and tested negative.

Commonly misidentified lines
(See ICLAC register)

No cell lines used in this study are listed in the Database of Cross-Contaminated or Misidentified Cell Lines (PMID 20143388).
